# Protective Effects of Mdivi‐1 on Cognition Disturbance Following Sepsis in Mice via Alleviating Microglia Activation and Polarization

**DOI:** 10.1111/cns.70149

**Published:** 2025-01-10

**Authors:** Chen Hong, Li Wang, Xiaowei Zhou, Liyong Zou, Xinming Xiang, Haoyue Deng, Qinghui Li, Yue Wu, Liangming Liu, Tao Li

**Affiliations:** ^1^ Shock and Transfusion Department, Research Institute of Surgery, Daping Hospital Army Medical University Chongqing China

**Keywords:** cognition, Mdivi‐1, mitochondrial fission, pyroptosis, sepsis‐associated encephalopathy

## Abstract

**Background:**

Neuroinflammation is one of the essential pathogeneses of cognitive damage suffering from sepsis‐associated encephalopathy (SAE). Lots of evidences showed the microglia presented mitochondrial fragmentation during SAE. This study investigated the protective effects and novel mechanisms of inhibiting microglia mitochondrial fragmentation via mitochondrial division inhibitor 1 (Mdivi‐1) on cognitive damage in SAE.

**Methods:**

The SAE model was performed by cecal ligation and puncture (CLP), and Mdivi‐1 was administrated via intraperitoneal injection. Morris water maze was performed to assess cognitive function. Mitochondrial morphology was observed by electron microscope or MitoTracker staining. The qRT‐PCR, immunofluorescence staining, and western blots were used to detect the inflammatory factors and protein content, respectively. Flow cytometry was used to detect the polarization of hippocampal microglia. Bioinformatics analysis was used to verify hypotheses.

**Results:**

Mdivi‐1 administration alleviated sepsis‐induced mitochondrial fragmentation, microglia activation, polarization, and cognitive damage. The mechanisms study showed neuroinflammation and oxidative stress were suppressed via NF‐κB and Keap1/Nrf2/HO‐1 pathways following Mdivi‐1 administration; meanwhile, pyroptosis in microglia was reduced, which was associated with enhanced autophagosome formation via p62 elevation following Mdivi‐1 administration.

**Conclusion:**

Inhibition of microglia mitochondrial fragmentation is beneficial to SAE cognitive disturbance, the mechanisms are related to alleviating neuroinflammation, oxidative stress, and pyroptosis.

## Graphical abstract

Inhibition of microglia mitochondrial fragmentation by mitochondrial division inhibitor 1 (Mdivi‐1) is beneficial to SAE cognitive disturbance, the mechanisms are mainly related to alleviating neuroinflammation, oxidative stress, and pyroptosis through NF‐κB, Keap1/Nrf2/HO‐1 pathways, and p62 enhancement, respectively.

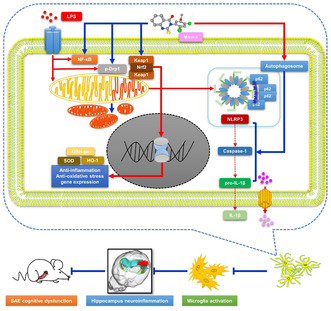

## 1 Background

Sepsis‐associated encephalopathy (SAE) refers to diffuse brain dysfunction associated with sepsis without central nervous system (CNS) infection, structural abnormalities, and other types of encephalopathy, which is one of the most common complications and sequelae of sepsis accounting for almost half of all sepsis patients [1, 2]. SAE not only increased morbidity of sepsis but also led to long‐term cognitive impairment. It is reported that 12.5%–21% of sepsis survivors suffered from cognitive disorders, especially involving learning and memory, which reduced the quality of life in survivors [3, 4]. Unfortunately, there are lack of specific treatments for SAE except for supportive treatments and antibiotic remedies [5, 6]. It is urgent to look for new treatments for cognitive dysfunction in SAE.

The pathogenesis of SAE is complicated and the mechanisms are still unclear [7]. However, neuroinflammation was testified to be the major mechanism of SAE [8, 9]. Microglia played a vital role in the development of neuroinflammation during SAE and are closely related to the cognitive dysfunction in sepsis [10, 11, 12, 13, 14].

A large of studies showed that mitochondrial function played a central and decisive role in cellular function [15]. Meanwhile, the mitochondrial structure determined the function of the highly dynamic organelles [16, 17]. Previous studies found that macrophage polarization was closely related to mitochondrial function, including mitochondrial metabolism disorder, cristae impairment, and mitochondrial permeability transition pore (mPTP) opening, the increase of mtDNA, and so on during inflammatory diseases [15, 18, 19]. Thus, mitochondrial dynamic determined cristae organization, mitochondrial metabolic models, mPTP, etc. and then dictated the mitochondrial function and cell fate. Previous studies also found that mitochondrial fragmentation was always characterized by following microglia polarization during the neuroinflammatory process [20]. Our previous studies demonstrated that macrophages exhibited mitochondrial fragmentation significantly during sepsis [21]. These above results indicated that inhibition of mitochondrial fragmentation may relieve neuroinflammation and cognitive disturbance following SAE.

Mdivi‐1, as a quinazolinone, was first identified as the selective inhibitor of Drp1 via a high‐throughput screen of small molecules [22]. So far, Mdivi‐1 has always been the most widely used and effective inhibitor of Drp1, which decreases Drp1‐mediated mitochondrial fission [23]. Meanwhile, Mdivi‐1 is considered to be a promising drug in the treatment of neurodegenerative diseases [24].

Herein, the aim of the present study was to observe the protective effects of Mdivi‐1 on the cognition decline of SAE mice, and the relationship between Mdivi‐1 and microglial polarization, neuroinflammation, oxidative stress, and pyroptosis were also investigated.

## 2 Materials and Methods

### 2.1 Drugs and Reagents

Mdivi‐1 was obtained from Tocris Bioscience (American, Cat. No. 3982). Lipopolysaccharide (LPS) was purchased from Sigma (St. Louis, MO, United States, L2630‐100 mg). Bafilomycin A1 was purchased from Selleck (S1413).

### 2.2 Establishment of CLP‐Induced SAE Mice Model and Experimental Design

The 8‐week‐female C57BL6J mice were obtained from the Animal Center, Daping Hospital, Army Medical University. Mice were fed in a cage with a humidity of 40% and a temperature of 24°C ± 1°C and light cycled (6 a.m.–6 p.m.). CLP was made as our previous article [21]. Animals were randomly divided into four groups: sham, sham + Mdivi‐1, CLP + vehicle, and CLP + Mdivi‐1. Mdivi‐1 was dissolved by DMSO and intraperitoneal injected in a dose of 20 mg/kg (Figure S1A).

### 2.3 Survival Rate, Murine Sepsis Score (MSS), and Neurobehavioral Score

After sepsis induction, mice of each group were followed hours through 48 h to establish the survival rate. Murine sepsis score was carried out to assess the severity of sepsis mainly based on reports by Mele and Sulzbacher [25, 26]. Neurobehavioral changes in mice were observed and scored 24 h after CLP to clarify neurobehavioral changes based on the reports of Zhao [27].

### 2.4 Morris Water Maze Measurement

Protocols were based on Charles V Vorhees and reported by our previous article [28, 29]. EthoVision XT 11.5 software was used for tracking and data collection.

### 2.5 Flow Cytometry

The protocol of the isolation of hippocampal microglia and flow cytometry was mainly from the reports of Qi Wan, Astrid E. Cardona, and Ye Xiong with minor adjustments [30, 31, 32]. Briefly, mice were anesthetized by 3% isoflurane and perfused by 0.9% saline and then the hippocampus were harvested immediately (8 mice per one sample). The hippocampus was minced and enzymatically dissociated with 0.5 mg/mL collagenase type III (Solarbio D6430), 1 mg/mL Dispase II (Beyotime Biotechnology, ST2339), and 1 mg/mL DNase I (BBI Life Sciences, A610099) in RPMI‐1640 (Gibco, C11875500BT) for 40 min at 37°C. The tissue fragment was centrifuged and filtered (100 μm) and resuspended with 30% Percoll (RPMI‐1640) layering over 70% Percoll (1 × PBS) and then centrifuged at 367g (2000 rpm) for 30 min at 4°C. The cells were collected from the interface of 70% to 30% Percoll solution, washed with PBS, and filtered (40 μm). The isolated cells were then stained with fluorochrome‐conjugated antibodies of CD45 (diluted by 1:400), CD11b (1:200), CD86 (1:100), CD68 (1:100), and CD206 (1:100) for 30 min and were washed with PBS and used for flow cytometry via BD FACSymphony A1 and then analyzed by the software FlowJo 10.8.1 [32]. The antibodies were all purchased from the BioLegend.

### 2.6 Transmission Electronic Microscopy Imaging

Fresh hippocampal tissues were prepared according to the previous article and observed with the transmission electron microscope (H‐7500, Hitachi Company, Japan) [33].

### 2.7 The Experimental Design of LPS‐Induced BV2 Cell Model

The BV2 cell line was divided into five groups: control, DMSO, Mdivi‐1, LPS + DMSO, and LPS + Midivi‐1. The Mdivi‐1 (10 nM) and the LPS (100 ng/mL) were co‐incubated for 24 h in our model (Figure S1B).

### 2.8 Immunofluorescence

The protocol was reported by our previous article [21]. The primary antibodies were as follows (diluted by 1:200): Iba‐1 (ab178846, Abcam), iNOS (ab49999, Abcam), CD206 (ab64693, Abcam), and NLRP3 (15101S, Cell Signaling Technology).

### 2.9 Quantitative Real‐Time PCR (qRT‐PCR) and Western Blots

These protocols were reported elsewhere. Primers and antibodies (diluted by 1:1000) were provided in Tables S1 and S2, respectively. The bands of Western blots were analyzed by ImageJ.

### 2.10 Adenovirus‐Mediated Overexpression and Knockdown of p62 in BV2 Cell

The sequence 5′‐GAGACGATGACTGGACACATT‐3′ was used to construct the *p62* shRNA adenovirus and mouse *p62* sequences (Gene ID: 18412) were used to construct *p62*‐expressing adenovirus from Shanghai Genechem Co. Ltd. The cells were infected with multiplicity of infection in a value of 20–30 for 6 h in DMEM basic without FBS and then for experimental use.

### 2.11 Detection of Oxidative Stress Indexes

The glutathione peroxidase (GSH‐px) and superoxide dismutase (SOD) were used as the oxidative stress indexes and detected by the corresponding kits (Beyotime Biotechnology, China, S0056, S0101M).

### 2.12 Mitochondrial Morphology Observation

The BV2 cell was inoculated in a confocal culture dish and grew to 70% fusion and then used for experiments. The MitoTracker Green FM was diluted in 1:5000. The mitochondrial branch length was measured by ImageJ [34].

### 2.13 Statistical Analysis

The two‐tailed Student's *t*‐test was performed to compare two groups. The one‐way ANOVA followed by Fisher's least significant difference post hoc or two‐way followed by Tukey's multiple comparisons test analysis was performed to compare multiple groups. The Kruskal–Wallis's test was performed to compare the frequency of crossing the platform among multiple groups. Shapiro–Wilk test for normality to assess data distribution. Statistical tests were performed using the GraphPad Prism 9 software. All differences among and between groups were statistically significant at *p* < 0.05.

## 3 Results

### 3.1 The Microglia Mitochondrial Excessive Fission in Microglia Was Positive Correlated With Neuroinflammation After Sepsis

**FIGURE 1:**
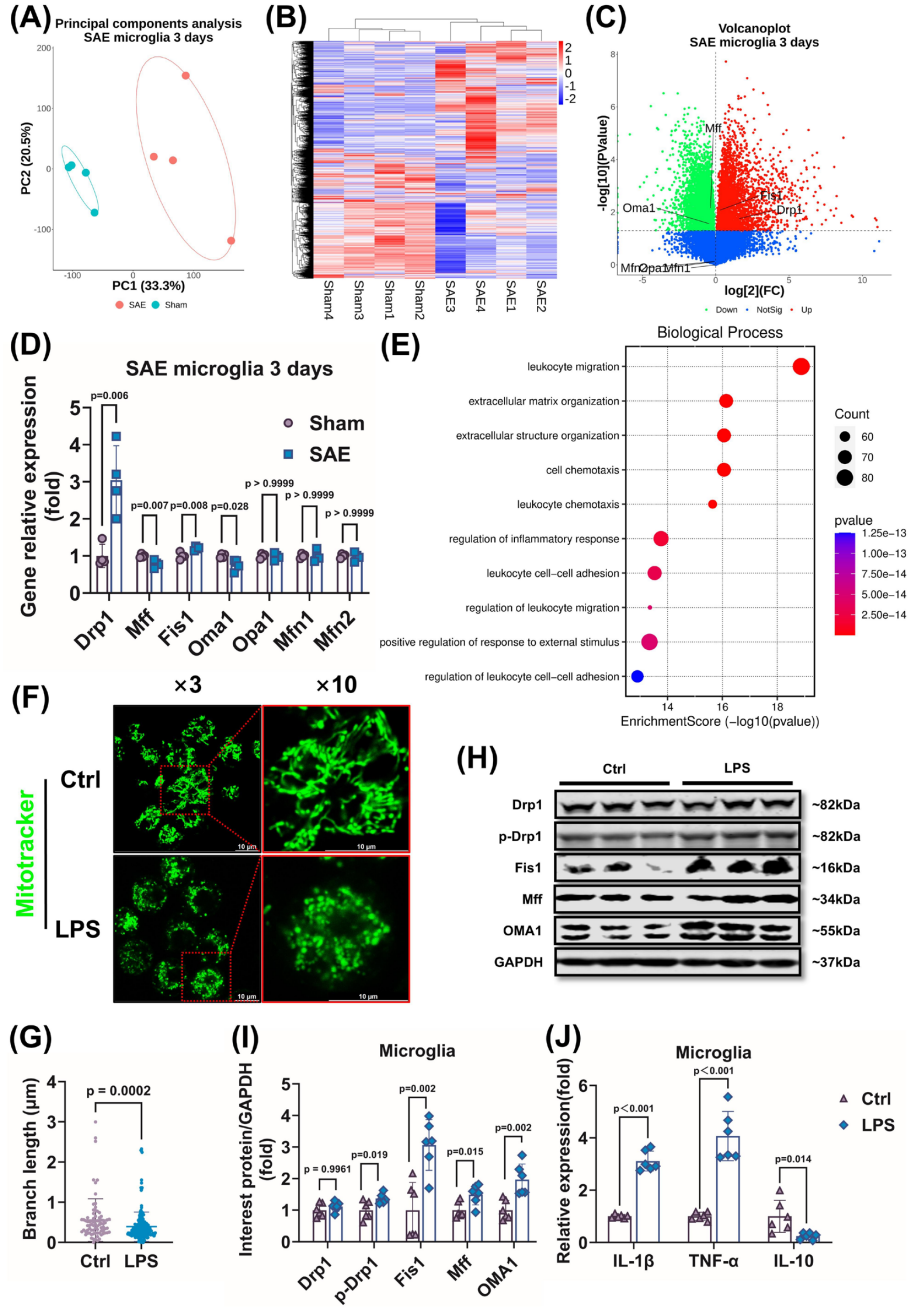
Microglia have excessive mitochondrial fission and neuroinflammation after sepsis both *in vivo* and *in vitro*. (A) Principal component analysis of gene expression in microglia after 3 days of sepsis (*n* = 4). (B) The differentially expressed genes were displayed in a heat map of microglia after 3 days of sepsis. (C) The volcanic map with gene tagging in microglia after 3 days of sepsis. (D) The mRNA relative expression of mitochondrial fusion and fission genes in microglia after 3 days of sepsis (*n* = 4). (E) The cluster analysis of the microglia after 3 days of sepsis. (F and G) Representative images (F) and mitochondrial branch length analysis (G) of microglia MitoTracker staining after 24 h of LPS‐induced BV2 cell model (*n* = 6). (H, I) Representative Western blots (H) and quantitative analysis (I) of Drp1, p‐Drp1, Fis1, Mff, and OMA1 of microglia after 24 h of LPS‐induced BV2 cell model. (J) The mRNA expression of inflammatory factors of *IL‐1β*, *TNF‐α*, and *IL‐10* in the LPS‐induced BV2 cell model. (H–J) *n* = 6 per group. Scale bar = 10 μm. All data were shown as mean ± SD.

To clarify the role of microglia mitochondrial fission in SAE, the data from transcriptome analysis of microglia at days 3 and 20 following sepsis induction (GSE198862) in the GEO data sets were analyzed to observe different expression genes in microglia. The results showed that gene expression in microglia from septic mice was distinct compared with the sham group (Figure 1A,B). Mitochondrial fission‐related gene expression including *Drp1*, *Fis1*, *Oma1*, and *Mff* was altered significantly, while the changes in gene expression mediating mitochondrial fusion, such as *Mfn1*, *Mfn2*, and *Opa1*, were not obvious (Figure 1C,D). Meanwhile, lots of inflammatory response genes were enriched in the result (Figure 1E) and microglia activation persists till 20 days (Figure S2).

To testify the results of data analysis, the mitochondrial morphology and related protein expression were observed in the BV2 cell. Results showed that the mitochondria in microglia presented excessive division and the branch length of mitochondrial was significantly shortened after LPS incubation (Figure 1F,G); Drp1 was activated by phosphorylation at Ser616 site, the levels of Fis1, OMA1, and Mff were also increased (Figure 1H,I). In the meantime, the inflammatory factors *IL‐1β* and *TNF‐α* were increased and anti‐inflammatory factor *IL‐10* was decreased (Figure 1J). These above results indicated that mitochondrial excessive fission was positively correlated with neuroinflammation in microglia after sepsis.

### 3.2 Mdivi‐1 Alleviated the Cognition Decline in SAE Mice

**FIGURE 2:**
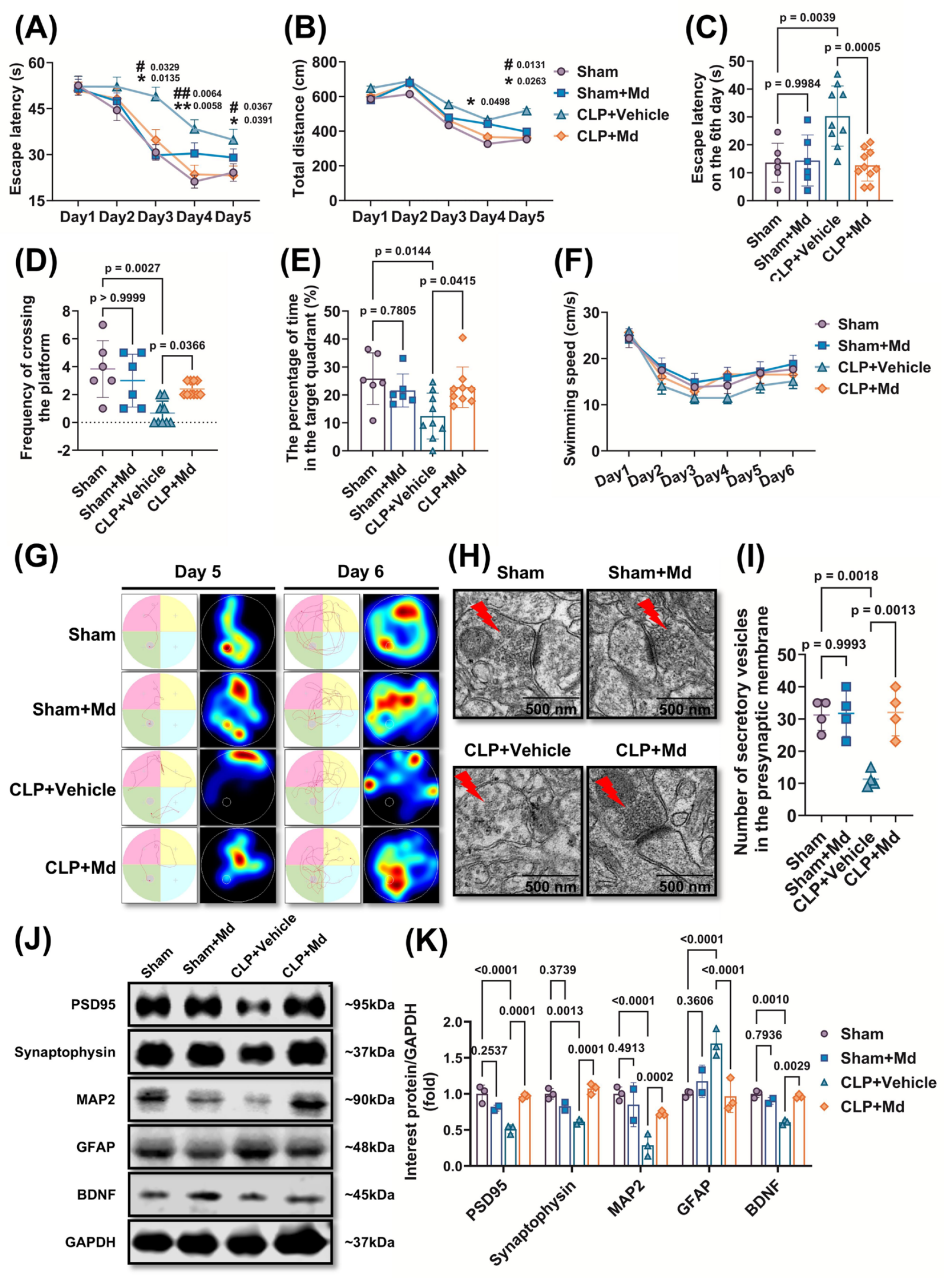
Mdivi‐1 relieves cognitive disturbance and protects synaptic structure in SAE mice. (A) Escape latency in spatial acquisition training (*F* = 98.47; *p* < 0.0001), (B) Total distance in spatial acquisition training (*F* = 404.9; *p* < 0.0001), (C) escape latency in probe trial (*F* = 8.828; *p* = 0.0003), (D) Frequency of crossing the platform in probe trial (K–W statistic = 14.93; *p* = 0.0019), (E) The percentage of time in the target quadrant in probe trial (*F* = 4.474; *p* = 0.0116), (F) swimming speed during trial (*F* = 0.3949; *p* = 0.9789), (G) Representative thermography and traces of mice movement on days 5 and 6, (H, I) Representative synaptic ultrastructure scanning by transmission electron microscope (H) and quantitative analysis (I) of synaptic vesicles in the hippocampus of SAE model mice after 3 months (*F* = 12.51; *p* = 0.0005), (J, K) Representative Western blots (J) and quantitative analysis (K) of PSD95, synaptophysin, MAP2, GFAP, and BDNF levels in the hippocampus of SAE mice after 3 months (*F* = 13.63; *p* < 0.0001), (A–F) Sham group (*n* = 6), Sham + Md group (*n* = 6), CLP + Vehicle group (*n* = 9), CLP + Md group (*n* = 10), (H, I) *n* = 2 per group, (J, K) Sham group (*n* = 3), Sham + Md group (*n* = 2), CLP + Vehicle group (*n* = 3), CLP + Md group (*n* = 3). In the picture A and B: *CLP + Vehicle group versus Sham group, ^#^CLP + Vehicle group versus CLP + Md group. Scale bar = 500 nm. The data in the A, B, and F were shown as mean ± SEM; the left data were shown as mean ± SD.

To observe the effects of mitochondrial fission inhibition on cognition of SAE, Mdivi‐1 (20 mg/kg) was administered after CLP. Compared with septic mice, the escape latency and total distance during the spatial acquisition test were alleviated after Mdivi‐1 administration (Figure 2A,B,G), and the frequency of crossing the platform and the percentage of time spent in the target quadrant were improved (Figure 2C–E,G). Swimming speed in all mice was comparable, which indicated that Mdivi‐1 did not have significant negative effects on mice motor functions (Figure 2A–F).

Moreovers, sepsis‐induced disturbances of synaptic ultrastructure and structural proteins were lightened after Mdivi‐1 administration, which were closely related to cognition in the hippocampus (Figure 2H–K). The neuron content, BDNF, and GFAP level in hippocampus were also recovered significantly after Mdivi‐1 administration. The results indicated that Mdivi‐1 protected sepsis‐induced neuronal damage and glial cell proliferation (Figure 2J,K). A further study showed that Mdivi‐1 improved the SAE‐induced neurobehavioral dysfunction and prolonged survival of septic mice (Figure S3). These above results suggested that Mdivi‐1 alleviated cognition disturbance following SAE.

### 3.3 Mdivi‐1 Reduced Microglia Activation and Corrected Microglia Polarization by Inhibiting Mitochondrial Excessive Fission

**FIGURE 3:**
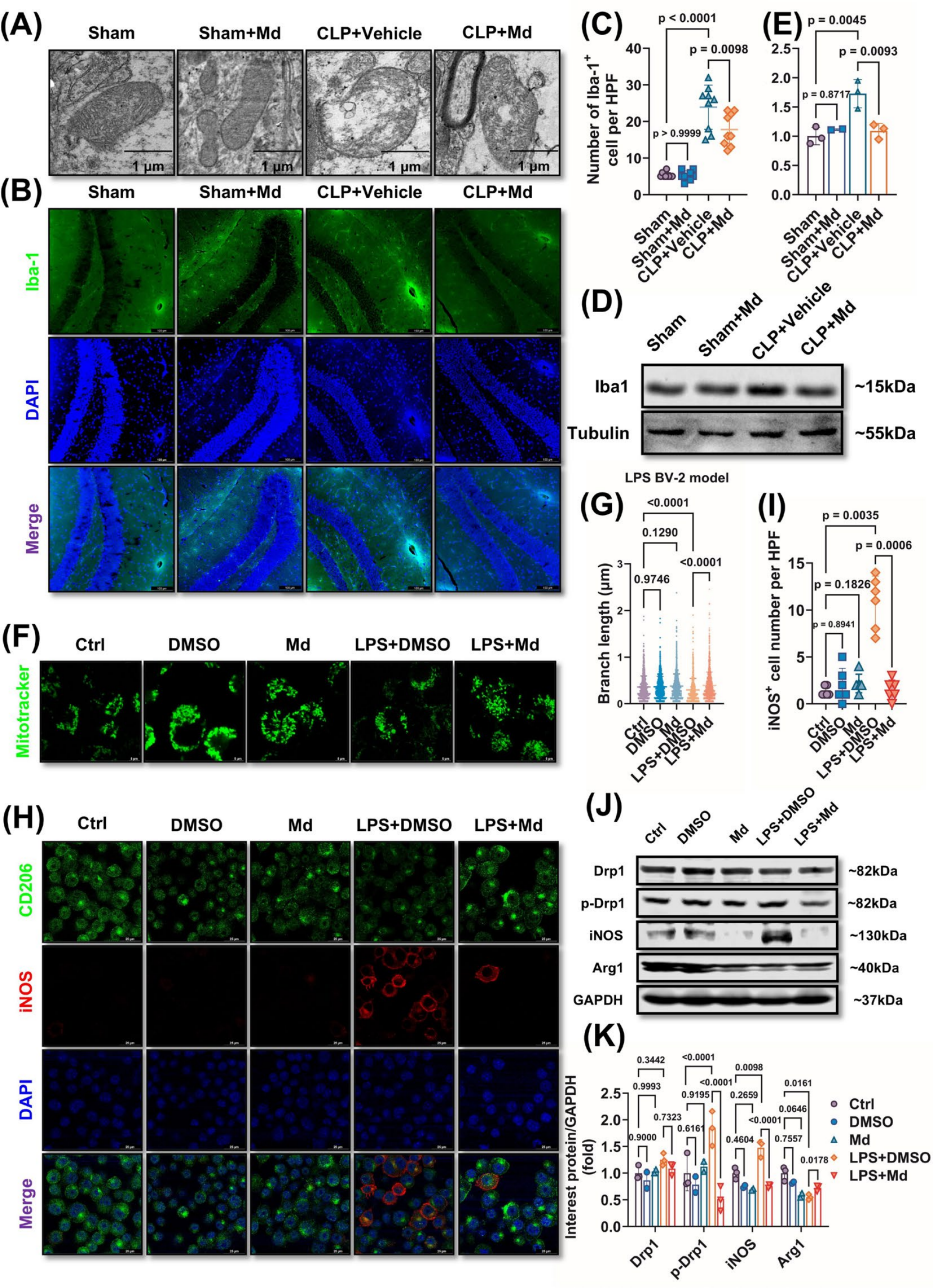
Mdivi‐1 protects hippocampal microglia mitochondrial morphology and regulates microglia polarization in sepsis. (A) Representative images of hippocampal microglia mitochondrial morphology scanning by transmission electron microscope of SAE mice after 12 h (scale bar = 1 μm). (B, C) Representative of immunofluorescence staining images of Iba‐1 (B) and Iba‐1 positive cell number qualitative analysis (C) of laser scanning confocal microscopy assay in the hippocampus of SAE mice after 12 h (Scale bar = 100 μm) (*F* = 53.24; *p* < 0.0001). (D, E) Representative Western blots (D) and quantitative analysis of Iba‐1 (E) of hippocampus in SAE mice after 12 h (*F* = 11.84; *p* = 0.0039). (F, G) Representative of Mito‐Tracker staining images (F) and mitochondrial branch length analysis (G) in LPS‐induced BV2 cell model (Scale bar = 5 μm) (*F* = 21.41; *p* < 0.0001). (H, I) Representative immunofluorescence staining images of CD206 and iNOS (H) and iNOS‐positive cells number analysis (I) of laser scanning confocal microscopy assay in LPS‐induced BV2 cell model (*F* = 42.31; *p* < 0.0001). (J, K) Representative Western blots (J) and quantitative analysis (K) of Drp1, p‐Drp1, Iba‐1, iNOS, and Arg1 levels in LPS‐induced BV2 cell model (*F* = 18.09; *p* < 0.0001). (A) *n* = 2 per group. (B, C, F–I) *n* = 3 per group. (D, E) Sham group (*n* = 3), Sham + Md group (*n* = 2), CLP + Vehicle group (*n* = 3), CLP + Md group (*n* = 3). (J, K) Ctrl group (*n* = 3 per group), DMSO group (*n* = 2 per group), Md group (*n* = 2 per group), LPS + DMSO group (*n* = 3 per group), LPS + Md group (*n* = 3 per group). All data were shown as mean ± SD.

A large number of studies demonstrated that microglia‐related inflammation was the major mechanism for the occurrence of SAE. To explore the mechanisms of Mdivi‐1 protecting SAE, the mitochondrial morphology in microglia and microglia function were observed. The results showed that the mitochondria in hippocampal microglia presented rupture following sepsis. Mdivi‐1 administration recovered mitochondrial morphology (Figure 3A). Meanwhile, Mdivi‐1 also decreased the number of Iba1‐positive cells and the content of Iba1 in hippocampus, which meant that Mdivi‐1 inhibited hippocampal microglia activation (Figure 3B–E). The similar effects of Mdivi‐1 were also testified *in vitro* (Figure 3F,G,J,K). Further, LPS incubation led to microglia polarization which presented as the increase in the number of iNOS‐positive cells and iNOS expressions, the decrease in the number of CD206‐positive cells and Arg1 expression compared with the control group; Mdivi‐1 administration antagonized LPS‐induced microglia activation and polarization (Figure 3H–K). These results indicated that Mdivi‐1 recovered the mitochondrial morphology of hippocampal microglia and then reduced hippocampal microglia activation and polarization, which participated in the protection of SAE.

### 3.4 Mdivi‐1 Lightened Sepsis‐Induced Hippocampal Neuroinflammation and Oxidative Stress via NF‐κB and Keap1/Nrf2/HO‐1 Pathways

**FIGURE 4:**
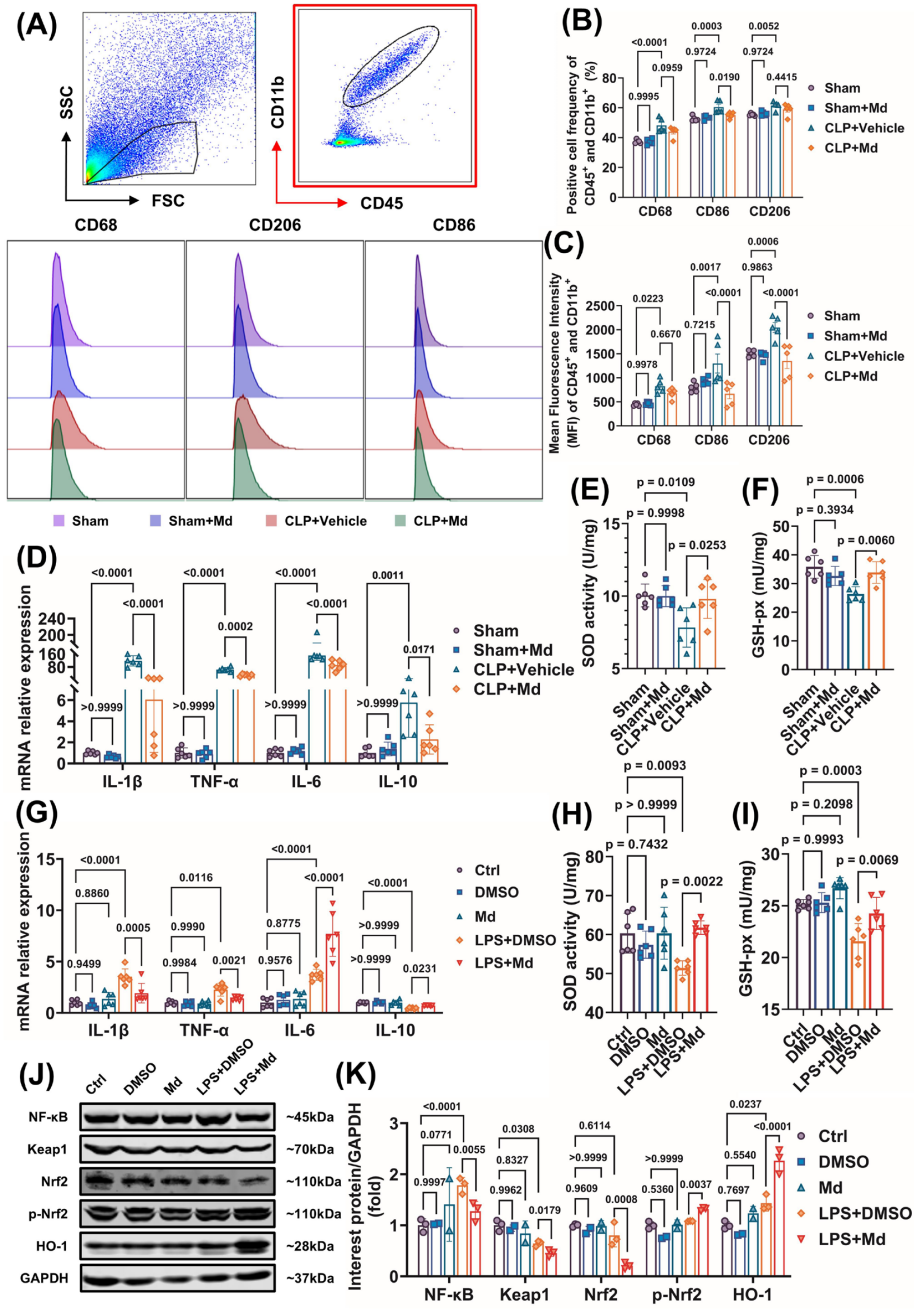
Mdivi‐1 inhibits hippocampal neuroinflammation and oxidative stress via NF‐κB and Nrf2/HO‐1 pathways. (A) Representative pseudo‐color and histograms of flow cytometry show the gating strategy for microglia (CD45^+^ CD11b^+^) and CD68^+^, CD86^+^, and CD206^+^ expressing microglia in the hippocampus suspensions from the sham and septic mice after 12 h of CLP. (B, C) The frequency of positive cells (B) and mean fluorescence intensity (MFI, C) of microglia (CD45^+^ CD11b^+^) in the hippocampus of the sham‐operated and septic mice after 12 h of CLP (B:F = 28.93; *p* < 0.0001; C:F = 20.06; *p* < 0.0001). (D) The mRNA expression of *IL‐1β*, *TNF‐α*, *IL‐6*, and *IL‐10* of hippocampus in SAE mice after 12 h (*F* = 200.1; *p* < 0.0001). (E, F) The oxidative stress indexes of SOD activity (E) and GSH‐px contents (F) of hippocampus in SAE mice after 12 h (E:F = 5.679; *p* = 0.0056; F:F = 8.489; *p* = 0.0008). (G) The mRNA expression of *IL‐1β*, *TNF‐α*, *IL‐6*, and *IL‐10* in LPS‐induced BV2 cell model (*F* = 47.60; *p* < 0.0001). (H, I) The oxidative stress indexes of SOD activity (H) and GSH‐px contents (I) in LPS‐induced BV2 cell model (H:F = 5.792; *p* = 0.0019; I:F = 14.47; *p* < 0.0001). (J, K) Representative of Western blots (J) and quantitative analysis (K) of NF‐κB, Keap1, Nrf2, p‐Nrf2, and HO‐1 in LPS‐induced BV2 cell model (*F* = 4.471; *p* < 0.0044). (A–C) *n* = 5; (D–I) *n* = 6 per group. (J, K) Ctrl group (*n* = 3 per group), DMSO group (*n* = 2 per group), Md group (*n* = 2 per group), LPS + DMSO group (*n* = 3 per group), LPS + Md group (*n* = 3 per group). All data were shown as mean ± SD.

Microglia activation resulted in inflammation and oxidative stress which was one of the major mechanisms of SAE. Therefore, the effects of Mdivi‐1 on inflammation and oxidative stress were observed *in vivo* and *in vitro*. The results showed that the polarization of hippocampal microglia (CD45^+^ CD11b^+^) toward M1 phenotype with the frequency or the mean fluorescence intensity (MFI) of CD68^+^, CD68^+^, and CD206^+^ cells increased after sepsis significantly (Figure 4A–C); meanwhile, the mRNA of *IL‐1β*, *TNF‐α*, *IL‐6*, and *IL‐10* were also significantly increased, whereas the SOD and GSH‐px were decreased after sepsis (Figure 4D–F), which was resumed by Mdivi‐1 administration *in vivo* (Figure 4A–F). Besides, the mRNA of *IL‐1β* and *TNF‐α* were significantly increased, and the levels of *IL‐10*, SOD, and GSH‐px were decreased after LPS incubation which were also resumed by Mdivi‐1 *in vitro* (Figure 4G–L).

The mechanisms of Mdivi‐1 alleviating inflammation and oxidative stress were further investigated *in vitro*. The results showed that NF‐κB activation by LPS incubation was inhibited by Mdivi‐1; and Mdivi‐1 incubation increased the levels of Nrf2 phosphorylation and its downstream molecules HO‐1 expressions and decreased the levels of Keap1 (Figure 4J,K). Collectively, these results indicated that Mdivi‐1 exerted the anti‐inflammation and antioxidant stress functions probably through NF‐κB and Keap1/Nrf2/HO‐1 pathways in the hippocampal microglia.

### 3.5 Mdivi‐1 Reduced Sepsis‐Induced Hippocampal Microglia Pyroptosis

**FIGURE 5:**
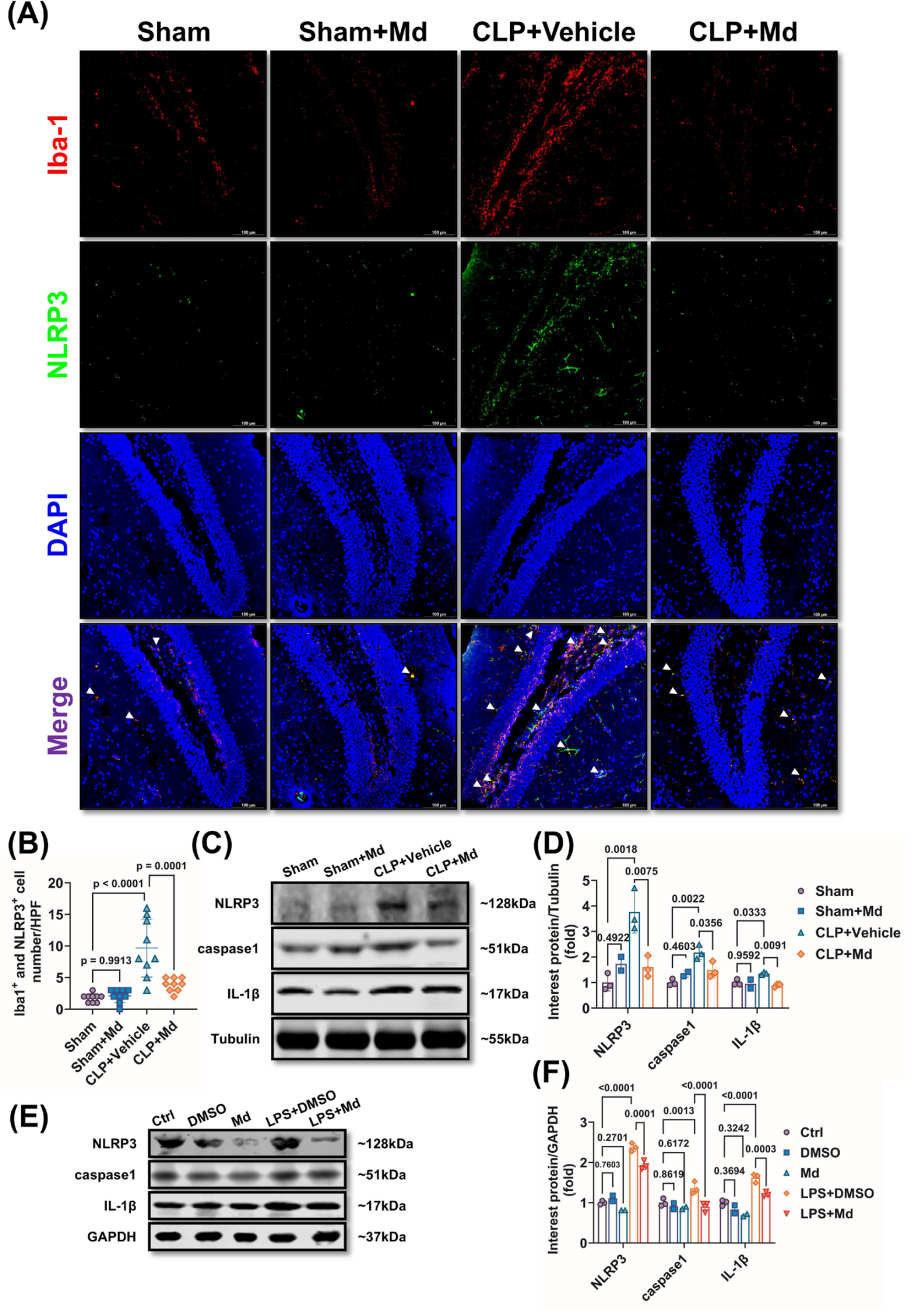
Mdivi‐1 suppresses hippocampal microglia pyroptosis. (A, B) Representative immunofluorescence staining images of Iba1 and NLRP3 (A) and double positive cell numbers analysis (B) of laser scanning confocal microscopy assay in SAE mice after 12 h (*F* = 20.34; *p* < 0.0001). (C, D) Representative Western blots (C) and quantitative analysis (D) of NLRP3, casapase‐1, and IL‐1β levels in hippocampus of SAE mice after 12 h (*F* = 28.52; *p* < 0.0001). (E, F) Representative Western blots (E) and quantitative analysis (F) of NLRP3, casapase‐1, IL‐1β levels in LPS‐induced BV2 cell model. (A, B) *n* = 3 per group (*F* = 128.5; *p* < 0.0001). (C, D) Sham group (*n* = 3), Sham + Md group (*n* = 2), CLP + Vehicle group (*n* = 3), CLP + Md group (*n* = 3). (E, F) Ctrl group (*n* = 3 per group), DMSO group (*n* = 2 per group), Md group (*n* = 2 per group), LPS + DMSO group (*n* = 3 per group), LPS + Md group (*n* = 3 per group). Scale bar = 100 μm. All data were shown as mean ± SD.

The previous studies demonstrated that pyroptosis in the immune cells played a vital role in cognitive dysfunction [35, 36]. Therefore, the effects of Mdivi‐1 on pyroptosis were tested both *in vivo* and *in vitro*. The results showed that NLRP3 content in hippocampal microglia was upregulated significantly in SAE mice, and Mdivi‐1 antagonized the phenomenon (Figure 5A–D). At the same time, the results also showed that the caspase‐1 and IL‐1β levels, the downstream molecules of pyroptosis, were decreased after Mdivi‐1 administration (Figure 5C,D). These above results were also observed *in vitro* (Figure 5E,F). These results indicated that Mdivi‐1 alleviated sepsis‐induced hippocampal microglia pyroptosis.

### 3.6 Mdivi‐1 Inhibited Microglia Pyroptosis Via Autophagosome Formation Promotion Through Improving p62 Level

Our previous studies showed that mitophagy was associated with pyroptosis and the effects of Mdivi‐1 on the autophagic flux were further detected [37]. The results showed that Mdivi‐1 enhanced LC3‐II transformation when coculture with Bafilomycin A1 (an autophagy inhibitor) (Figure S4A,B). Further, the AMPK was activated and mTOR was inhibited after LPS incubation, and Mdivi‐1 reversed those effects (Figure S4C,D). These results demonstrated that Mdivi‐1 promoted autophagosome formation and enhanced autophagic flow. Further studies showed that Mdivi‐1 administration only increased the level of *p62* significantly (Figure S4E,F). These above results implied that Mdivi‐1 might inhibit pyroptosis via promoting autophagosome formation associated with p62.

**FIGURE 6:**
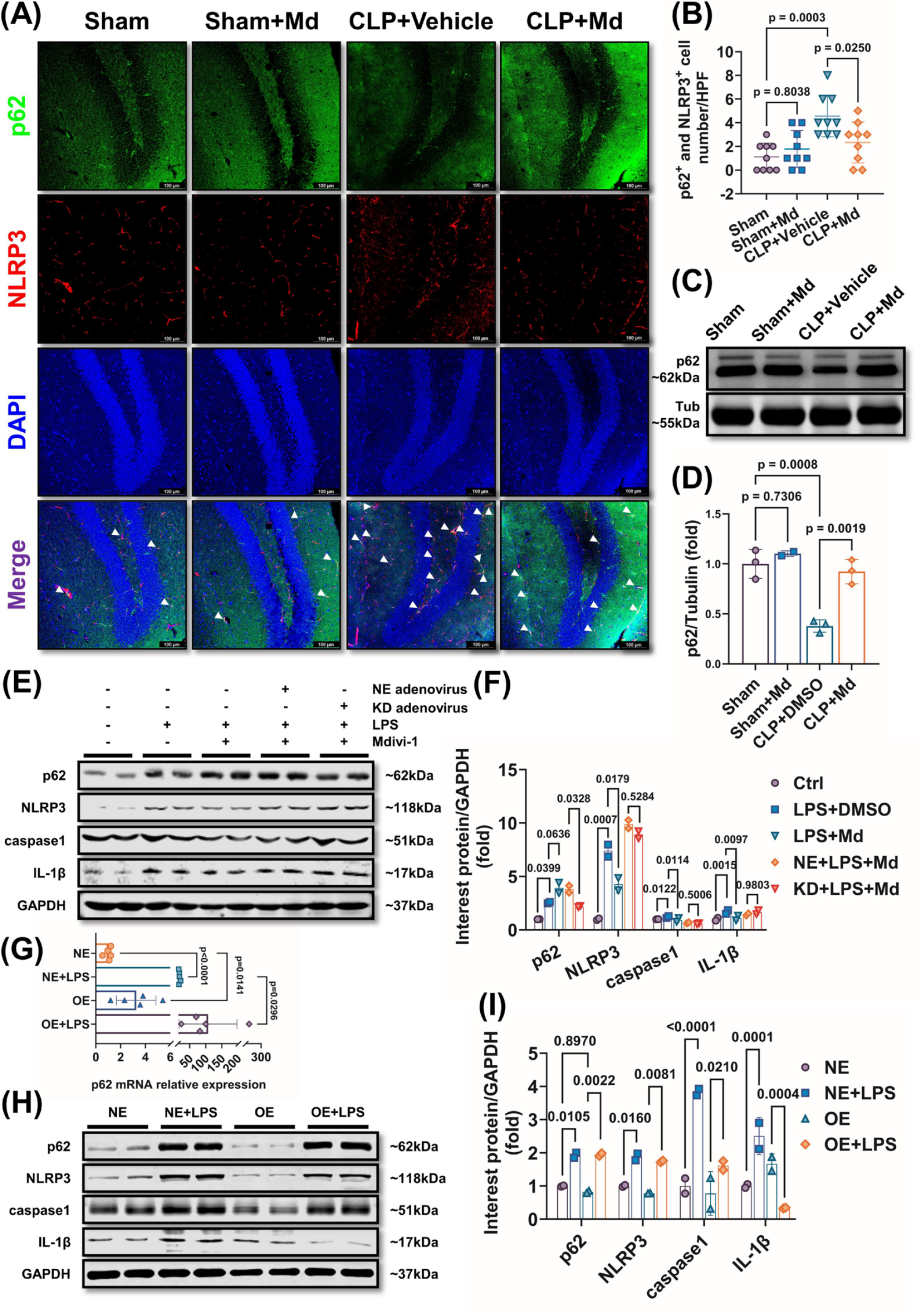
Mdivi‐1 inhibits hippocampal microglia pyroptosis through enhancing p62‐mediated autophagosome formation. (A, B) Representative of immunofluorescence staining images of NLRP3 and p62 (A) and NLRP3 and p62 double positive cells number analysis (B:F = 8.166; *p* = 0.0004) of laser scanning confocal microscopy assay in the hippocampus of SAE mice after 12 h. (C, D) Representative Western blots (C) and quantitative analysis (D) of p62 in the hippocampus of SAE mice after 12 h (*F* = 25.38; *p* = 0.0004). (E, F) Representative Western blots (E) and quantitative analysis (F) of p62, NLRP3, casapase‐1, IL‐1β levels in LPS‐induced BV2 cell model under *p62* knockdown condition (*F* = 28.85; *p* < 0.0001). (G) *p62* mRNA expression levels in LPS‐induced BV2 cell model under *p62* overexpression condition *in vitro* (*F* = 6.286; *p* = 0.0051). (H, I) Representative Western blots (H) and quantitative analysis (I) of p62, NLRP3, casapase‐1, IL‐1β levels in LPS‐induced BV2 cell model under *p62* overexpression condition (*F* = 14.11; *p* < 0.0001). (A, B) *n* = 3 per group. (C, D) Sham group (*n* = 3), Sham+Md group (*n* = 2), CLP + Vehicle group (*n* = 3), CLP + Md group (*n* = 3). (E, F) *n* = 2 per group. (G) *n* = 5 per group. (H, I) *n* = 2 per group. Scale bar = 100 μm. All data were shown as mean ± SD.

To further elucidate the relationship between p62 and pyroptosis in SAE, p62, and NLRP3 co‐staining was conducted, and we found that the numbers of p62‐NLRP3‐positive cells were higher after sepsis than the Sham group, whereas it was reduced after Mdivi‐1 administration *in vivo* (Figure 6A,B). Meanwhile, the content of p62 was decreased significantly in hippocampus after sepsis, and Mdivi‐1 reversed the decrease of p62 (Figure 6C,D). Further study showed that the inhibition effects of Mdivi‐1 on pyroptosis in microglia were antagonized by *p62* knockdown (Figure 6E,F). Meanwhile, *p62* overexpression by administrating the *p62* mRNA vector adenovirus could decrease the contents of NLRP3, caspase‐1, and IL‐1β (Figure 6H,I). These results demonstrated that Mdivi‐1 inhibited microglia pyroptosis by improving p62 level and autophagosome formation in microglia.

### 3.7 The Disorder of Mitochondrial Dynamics and Inflammation Were Testified in Peripheral Blood Mononuclear Macrophages in Septic Patients

To testify mitochondrial morphology disturbance in septic patients, the transcriptome analysis of blood monocytes from sepsis patients (GSE46955) was conducted. A lot of genes of mitochondria in the peripheral blood mononuclear macrophages were involved in sepsis patients (Figure S5A–E). The cluster analysis showed that genes‐related mitochondrial matrix, mitochondrial inner membrane, as well as secretion and phagocytosis were enriched in the cellular component (Figure S5D). Meanwhile, cell adhesion and immunity process altered in the cluster analysis of biological processes (Figure S5E). The above results suggested that mononuclear macrophages in sepsis patients undergo mitochondrial morphology and function disturbance, accompanied by significant variations in immune function. The results indicated that modulation of mitochondrial morphology and function in monocyte macrophages might become a potent target for treating inflammation‐related complications (e.g., SAE) in sepsis patients.

## 4 Discussion

In the present study, we found that Mdivi‐1 protected cognitive disturbance in SAE mice via inhibition of mitochondrial fission in microglia. The hippocampal microglia in septic mice presented obvious mitochondrial fission and neuroinflammation; Mdivi‐1 alleviated microglia activation and polarization during sepsis and then protected learning and memory damage in septic mice via correcting mitochondrial morphology. The anti‐neuroinflammation and antioxidative stress of Mdivi‐1 were mainly through NF‐κB and Keap1/Nrf‐2/HO‐1 pathways, respectively. Meanwhile, Mdivi‐1 also inhibited microglia pyroptosis by enhancing p62 expression with the promoted autophagosome formation. Our results provided a potential therapeutic target as well as a novel drug candidate for the treatment of cognition decline in SAE.

Neuroinflammation was demonstrated to be the major mechanism of SAE; and microglia, the brain‐specific macrophage in the CNS, played a vital role in the development of neuroinflammation during SAE, which is closely related to the cognitive dysfunction in sepsis [38, 39]. Previous studies found that macrophage polarization was regulated by mitochondrial metabolism during inflammatory diseases, which was closely related to mitochondrial fission, cristae impaired, and mPTP open [18, 19, 40, 41]. Meanwhile, our previous work also found macrophages exhibited mitochondrial fragmentation significantly during sepsis. In addition, Drp1, a kind of GTPase and mediated mitochondrial fission, was the direct key “executor” of mitochondrial fission [42]. Mdivi‐1, as a quinazolinone, was first identified as the selective inhibitor of Drp1 via a high‐throughput screen of small molecules [22]. So far, even though several small molecules or polypeptides, including elamipretide (SS‐31), dynasore, and P110, have been reported to have the inhibitory effects on Drp1, Mdivi‐1 has always been the most widely used and effective inhibitor of Drp1 [23]. Therefore, Mdivi‐1 is a promising drug in the treatment of neurodegenerative diseases for the role of reducing mtROS production, inflammation, and autophagy [24]. Our study demonstrated the protective effects of cognitive decline from microglia mitochondrial fragmentation by Mdivi‐1 after sepsis through alleviating microglia polarization and neuroinflammation probably via NF‐κB and Nrf2/Keap1/HO‐1. However, further exploration is needed to determine whether there are other mechanisms during these processes by using multi‐omics techniques. Meanwhile, although we had reported the protective effects of Mdivi‐1 on mitochondrial membrane potential, ATP production, ROS level, and mPTP function, our study still lacked the effects of Mdivi‐1 on microglia mitochondrial functions [43]. Therefore, we will focus on the relationship between mitochondrial morphology and function as well as the relationship between mitochondrial morphology and cellular function in the future, which is very meaningful and worth further exploration.

The inflammasome is the fundamental component of neuroinflammation and plays a vital role in the SAE, especially for the NLRP3 inflammasome, which is mainly expressed in microglia and astrocytes; besides, recent studies have demonstrated that SAE neuroinflammation is dependent on the NLRP3 in microglia specifically [44]. Our study found that Mdivi‐1 alleviated sepsis‐induced microglia NLRP3 through enhancing p62 level, which mediating autophagosome formation. However, drawing on the findings of Yang et al. and Panicker et al. [45, 46], astrocytes and neurons also have NLRP3 activation in CNS diseases. Although microglia make up approximately 10% of CNS cells and are closely related to cognitive function, we still lack of the understanding of whether the Mdivi‐1‐mediated NLRP3/p62 pathway is unique in microglia by immunofluorescent colocalization stains, especially for that Mdivi‐1 has extremely strong lipid solubility [47, 48]. Therefore, we will focus on the effects of Mdivi‐1 on glia, neurons, blood–brain barrier, etc. by single‐cell‐sequencing technology in the further exploration to clarify the mechanism of Mdivi‐1 on various types of neurocytes.

In addition, as the immunocyte colonized in the CNS, microglial has the functions of immunosurveillance and synaptic pruning, which will cause damage to neurons and synaptic structures during the process of illness [49]. Yin et al. [50] demonstrated that the cognitive impairment was accompanied by neuron dysfunction mainly through aberrant synaptic pruning caused by microglial activation. They conducted the microglia phagocytic activity, HMGB1‐induced microglial activation, synapse engulfment, and electrophysiological experiments on the hippocampal neural circuit to prove the causal relationship between microglia, neuroinflammation, and cognition decline in SAE. Moreover, Zhang et al. [51] also demonstrated that SB‐3CT‐inhibited MMP‐9 rescued the abnormal hippocampal CA1 γ oscillations caused by PV interneurons in perineuronal net in the cognition decline of SAE. We found Mdivi‐1 alleviated hippocampal microglia activation and polarization and protected the hippocampal neurons and synapses structure in SAE, however, our study lacked direct evidence of protecting hippocampal neural circuit through protecting microglia mitochondrial morphology or neuroinflammation. Hence, we should pay attention to the causality among microglia, hippocampal neural circuits, and cognition in future studies.

Last but not the least, our reports dropped a hint that Mdivi‐1 regulated p62 level in microglia significantly. Considering p62 contains six multifunctional domains, it becomes a center of multiple signal pathways, such as Nrf2, NF‐κB, and mTOR, and participates regulation of numerous basic cellar functions, these results called us to mind the link of autophagy, Mdivi‐1, p62, and other cellar functions in microglia [52]. Another possibility is that Mdivi‐1 is a multi‐targets and multi‐mechanisms small molecule drug just like itaconate in the treatment of sepsis [53]. Therefore, whether p62 is the upstream key molecule after Mdivi‐1 administration still needs to be illuminated in the future [54, 55].

## 5 Conclusion

Mdivi‐1 alleviated hippocampal microglia mitochondrial excessive fission and suppressed its activation, polarization, neuroinflammation, oxidative stress, and pyroptosis, thus aberrating cognitive impairment in SAE. The mechanisms of Mdivi‐1 on the inhibiton of microglia polarization through increasing p62 and autophagosome formation as well as the NF‐κB and Keap1/Nrf2/HO‐1 pathways probably (Figure S6). Therefore, protecting microglia mitochondrial morphology in SAE might be a potential target, and Mdivi‐1 can be a novel drug candidate for the treatment of SAE cognitive disturbance.

## Author Contributions

C.H. performed most of the experiments and data analyses and wrote the paper. L.W., L.Z., X.Z., X.X., H.D., Q.L., and Y.W. performed some experiments and/or data analysis. L.L. and T.L. designed the study, performed data analysis, and revised the paper. All authors read and approved the final manuscript.

## Ethics Statement

All animal experiments and operations were approved by the Research Council and Animal Care and Use Committee of the Army Medical Center, Army Medical University (AMUWEC20237101).

## Conflicts of Interest

The authors declare no conflicts of interest.

## Supporting information


Data S1.

Appendix S1.

## Data Availability

The data that support the findings of this study are available from the corresponding author upon reasonable request.
