# Supplementary material for: Protective Effects of Mdivi‐1 on Cognition Disturbance Following Sepsis in Mice via Alleviating Microglia Activation and Polarization
Source: CNS Neurosci Ther. 2025 Jan 10;31(1):e70149. doi: 10.1111/cns.70149 (PMC11719124; doi:10.1111/cns.70149)

Full unedited gel/blot for Figure 1 G-1

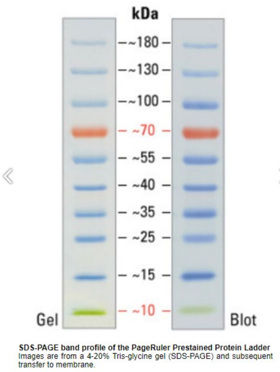

We used Thermoscientific PageRuler Prestained Protein Ladder (Ref 26616) for the protein molecular weight (~kDa) marker.

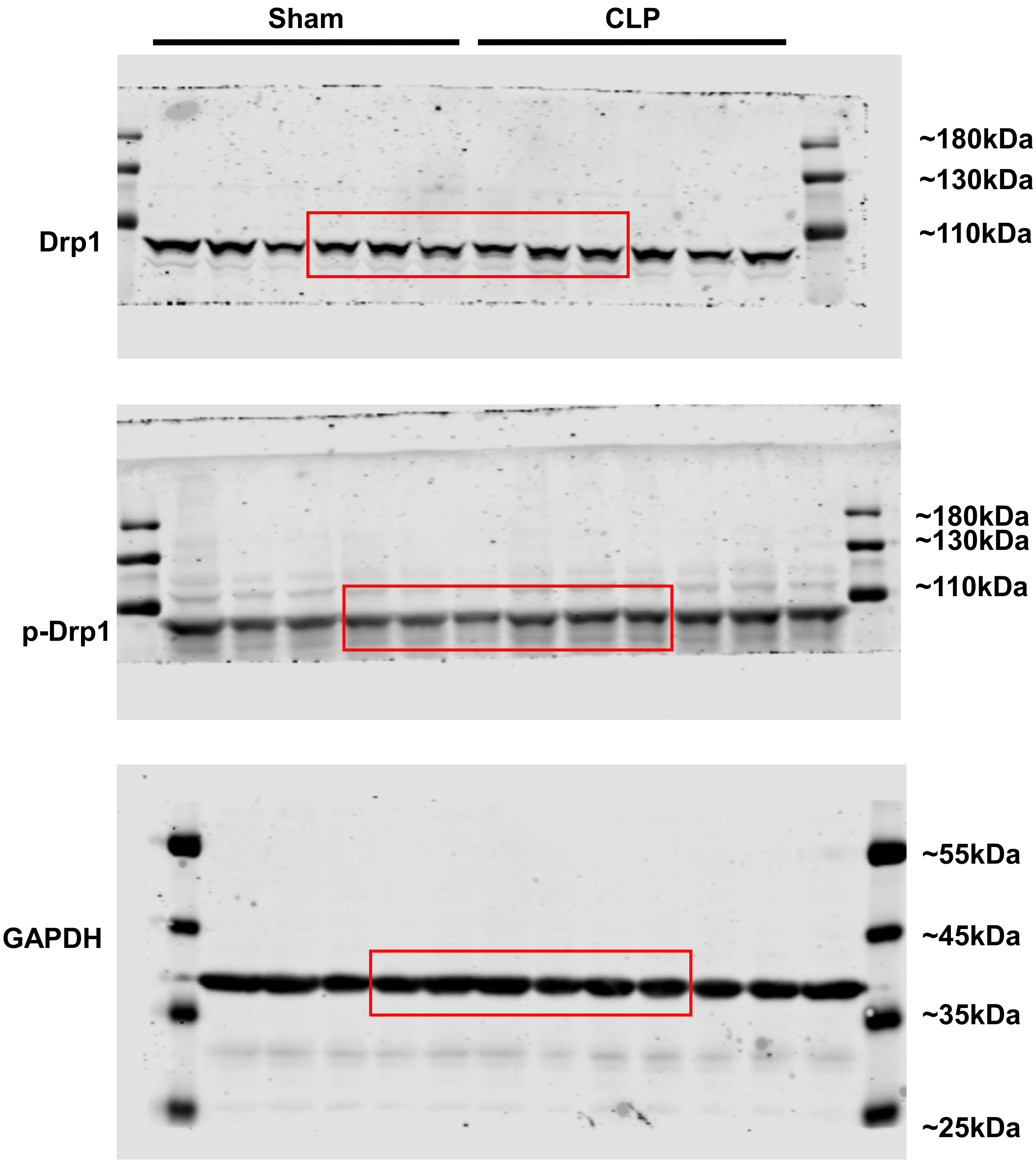

Full unedited gel/blot for Figure 1 G-2

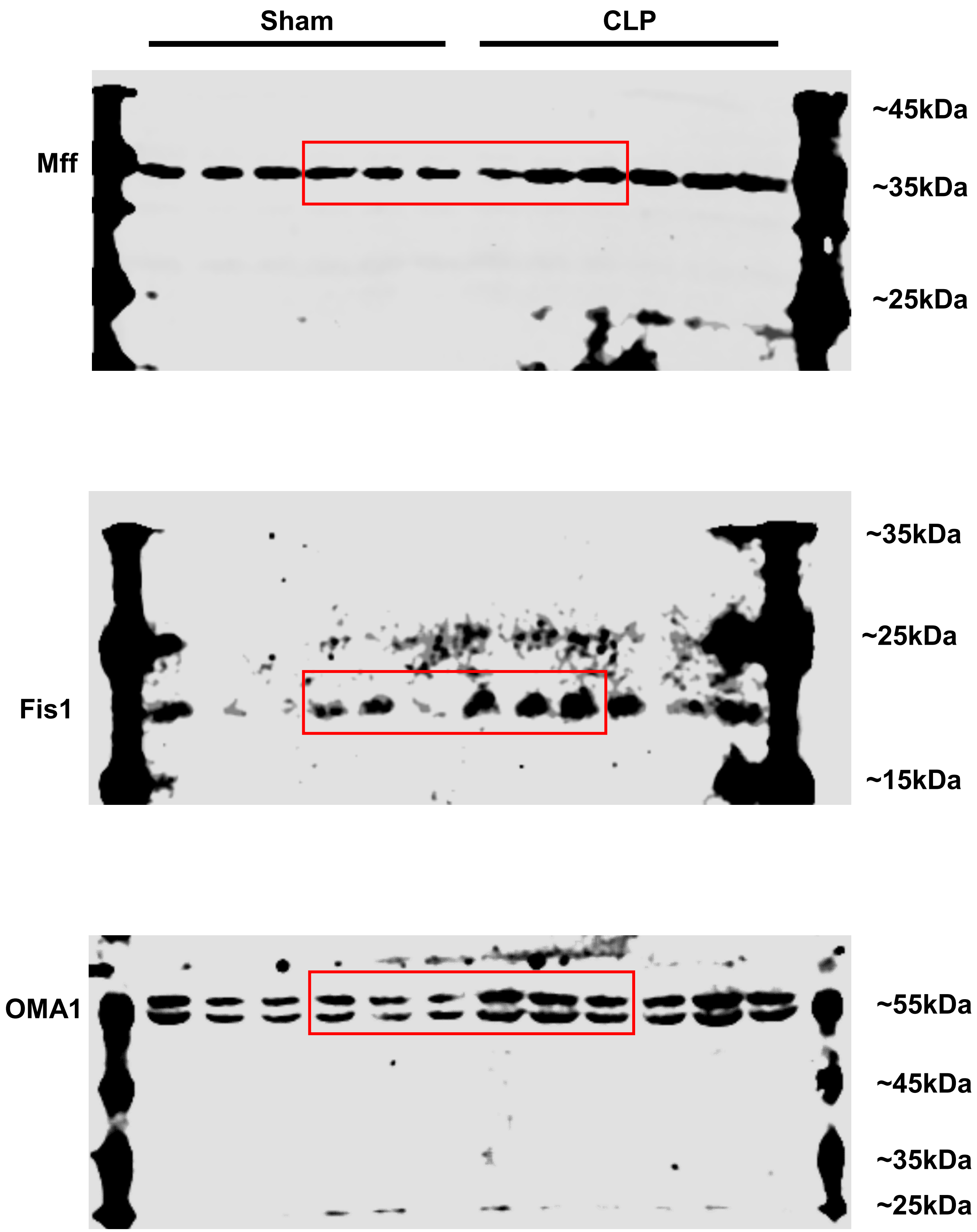

Full unedited gel/blot for Figure 2 J-1

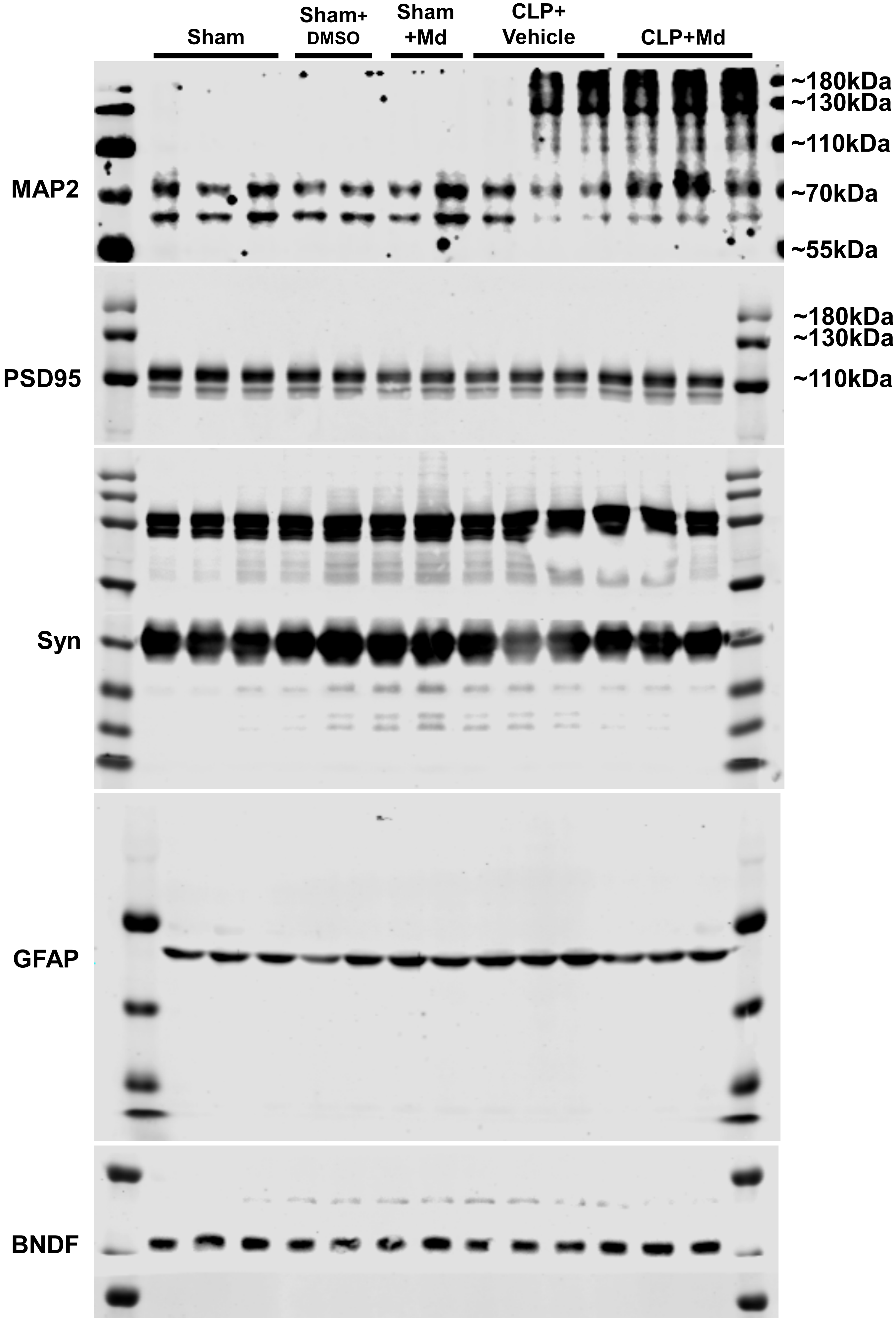

Full unedited gel/blot for Figure 2 J-2

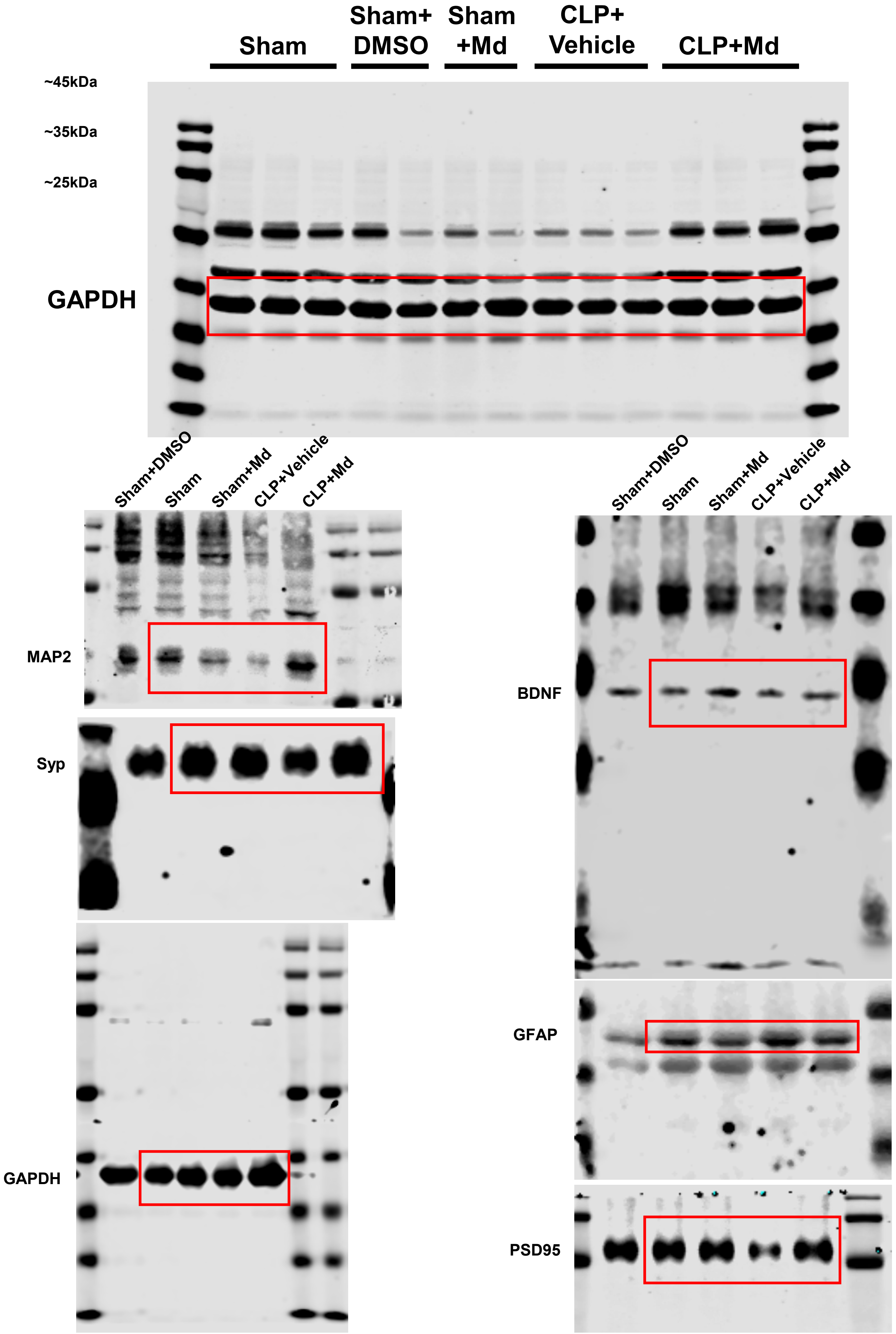

Full unedited gel/blot for Figure 3 D-1

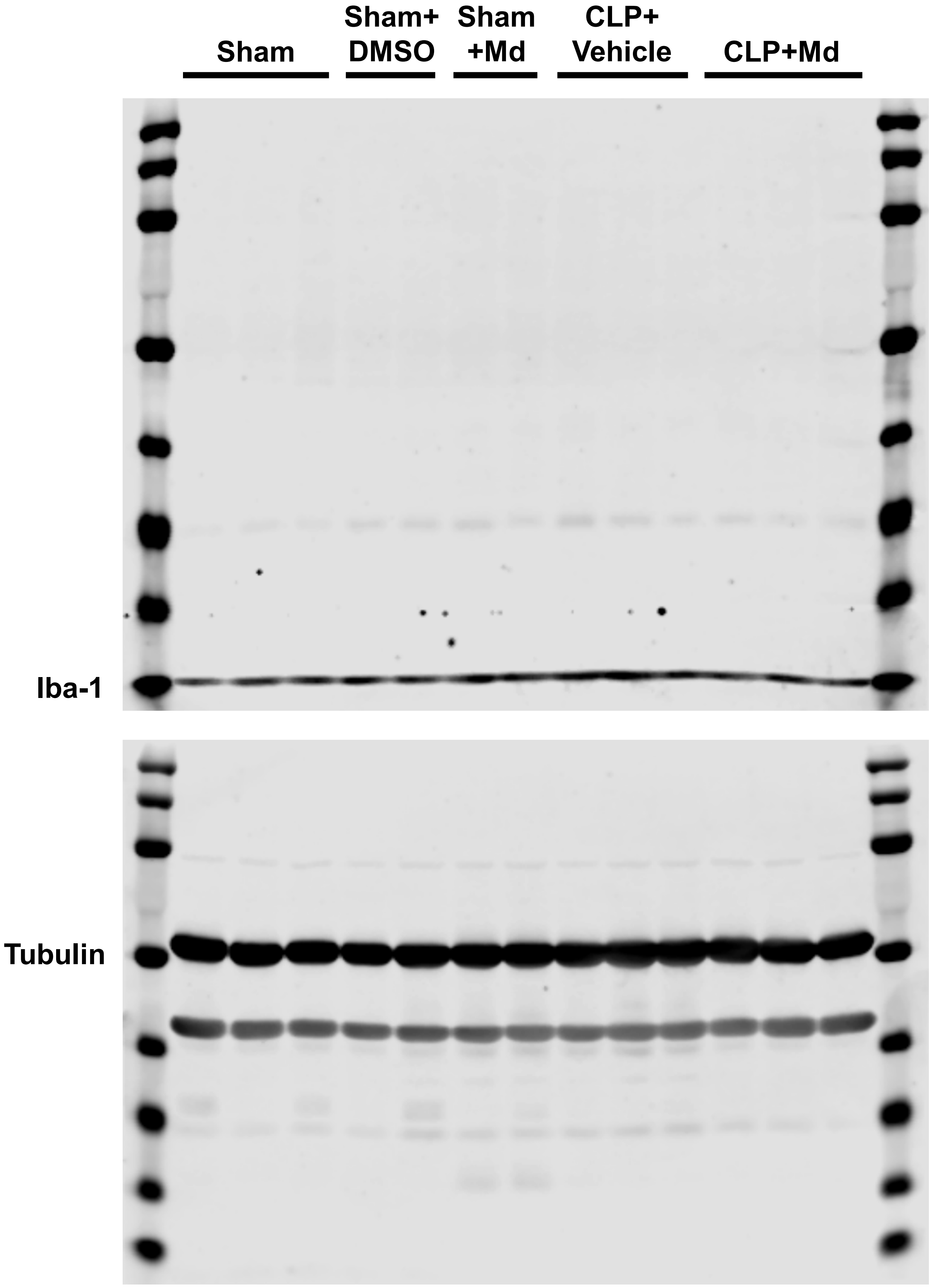

Full unedited gel/blot for Figure 3 D-2

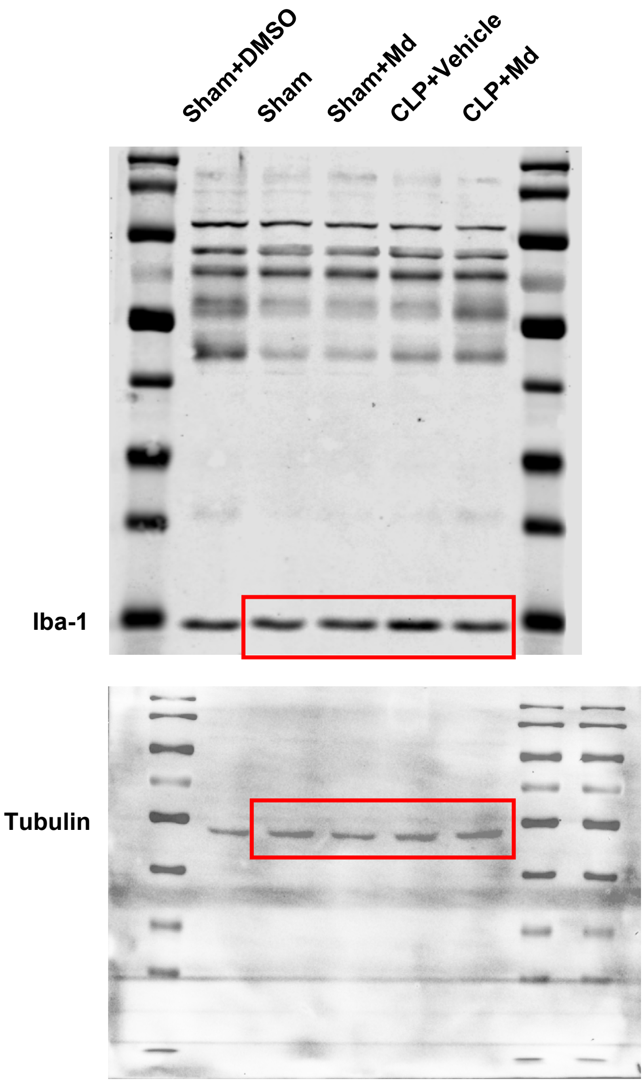

Full unedited gel/blot for Figure 3 J-1

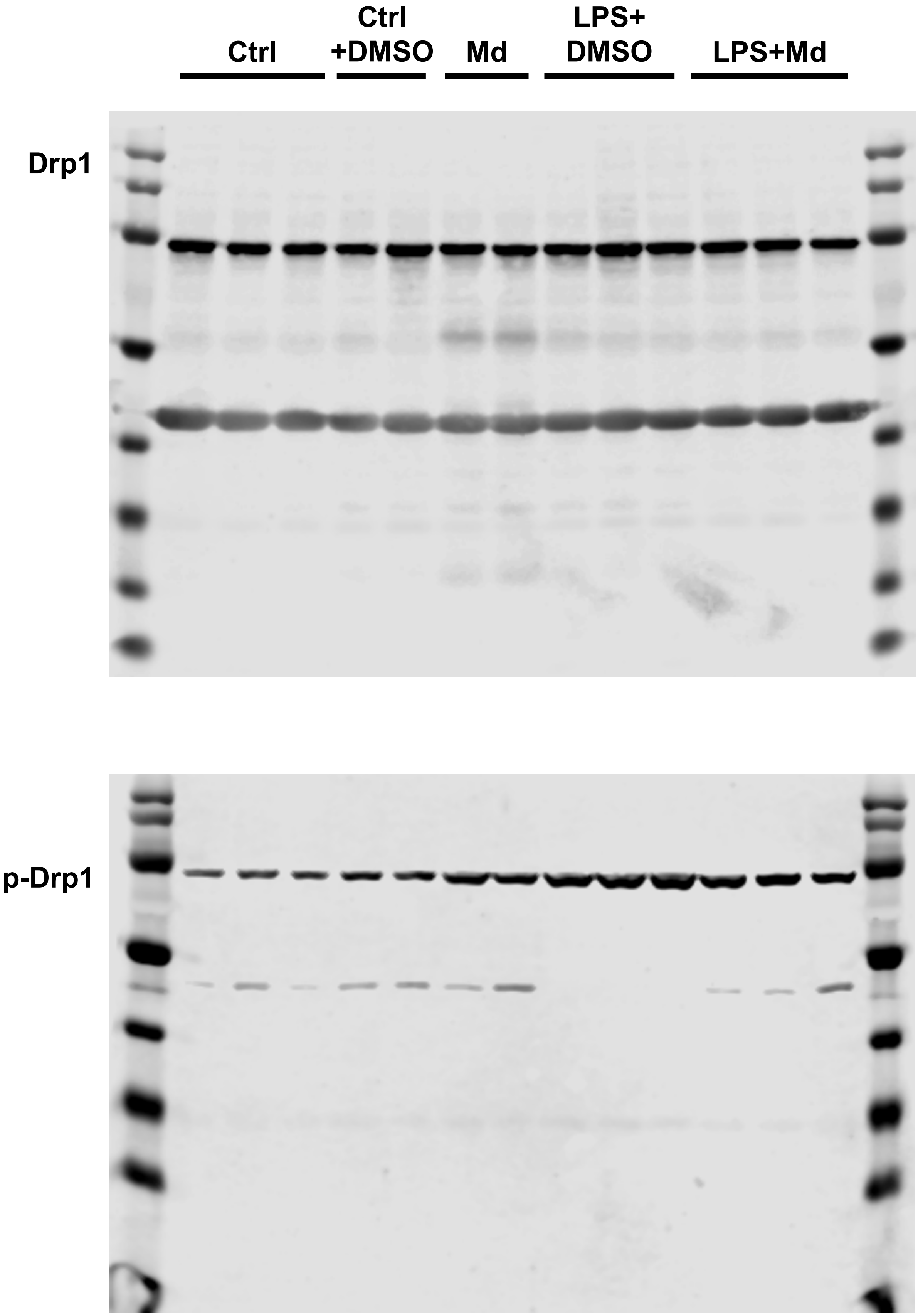

Full unedited gel/blot for Figure 3 J-2

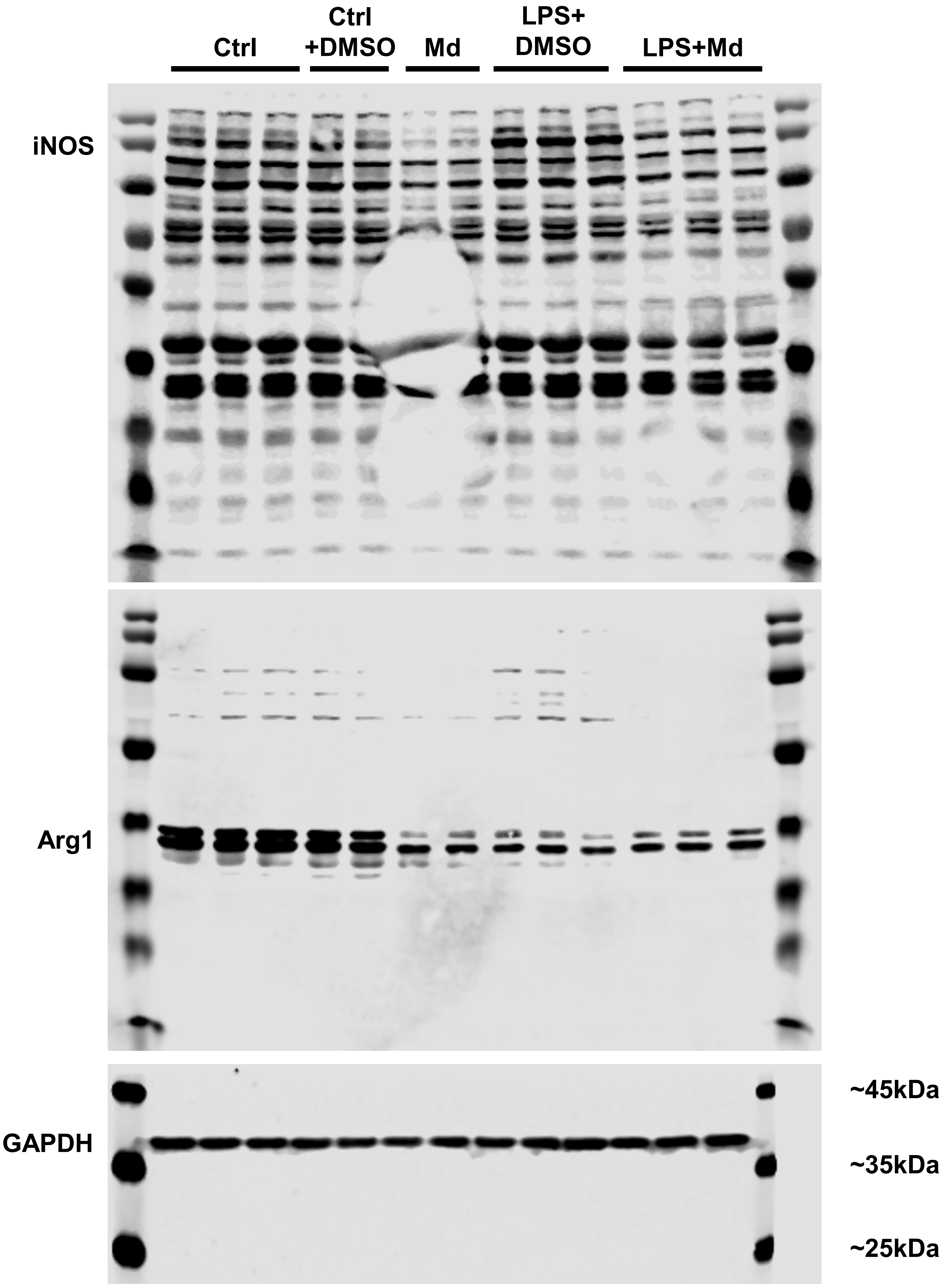

Full unedited gel/blot for Figure 3 J-3

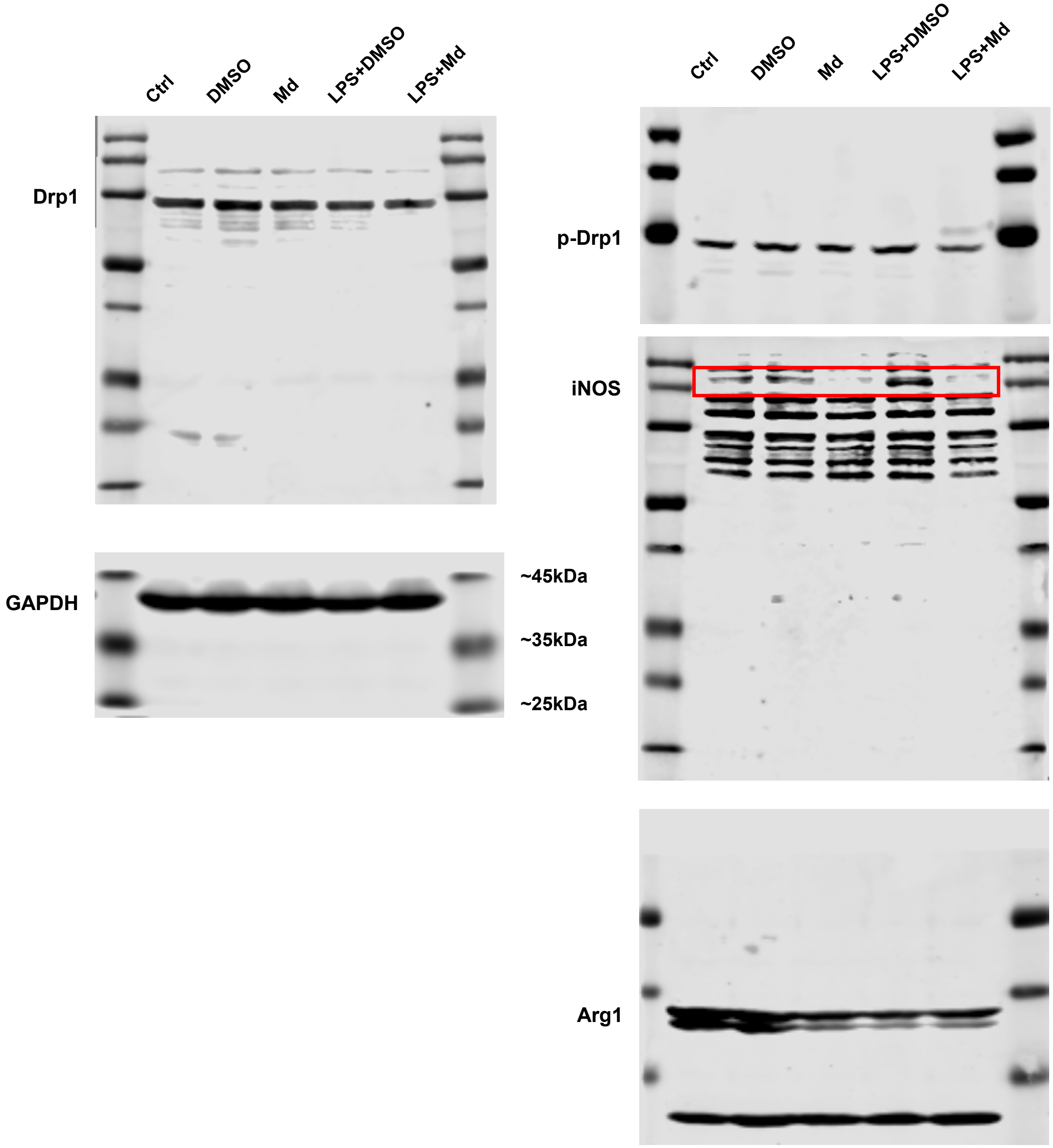

Full unedited gel/blot for Figure 4 J-1

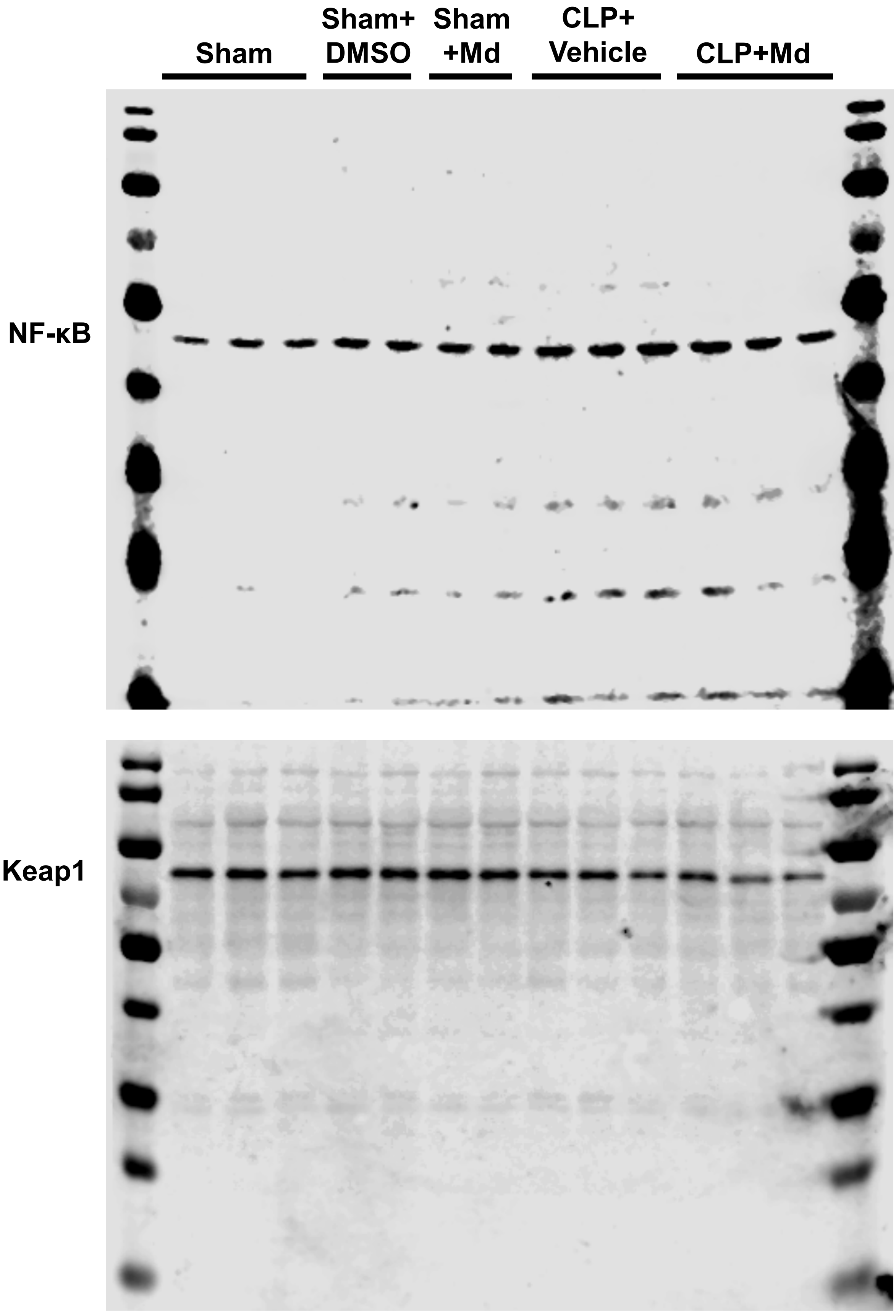

Full unedited gel/blot for Figure 4 J-2

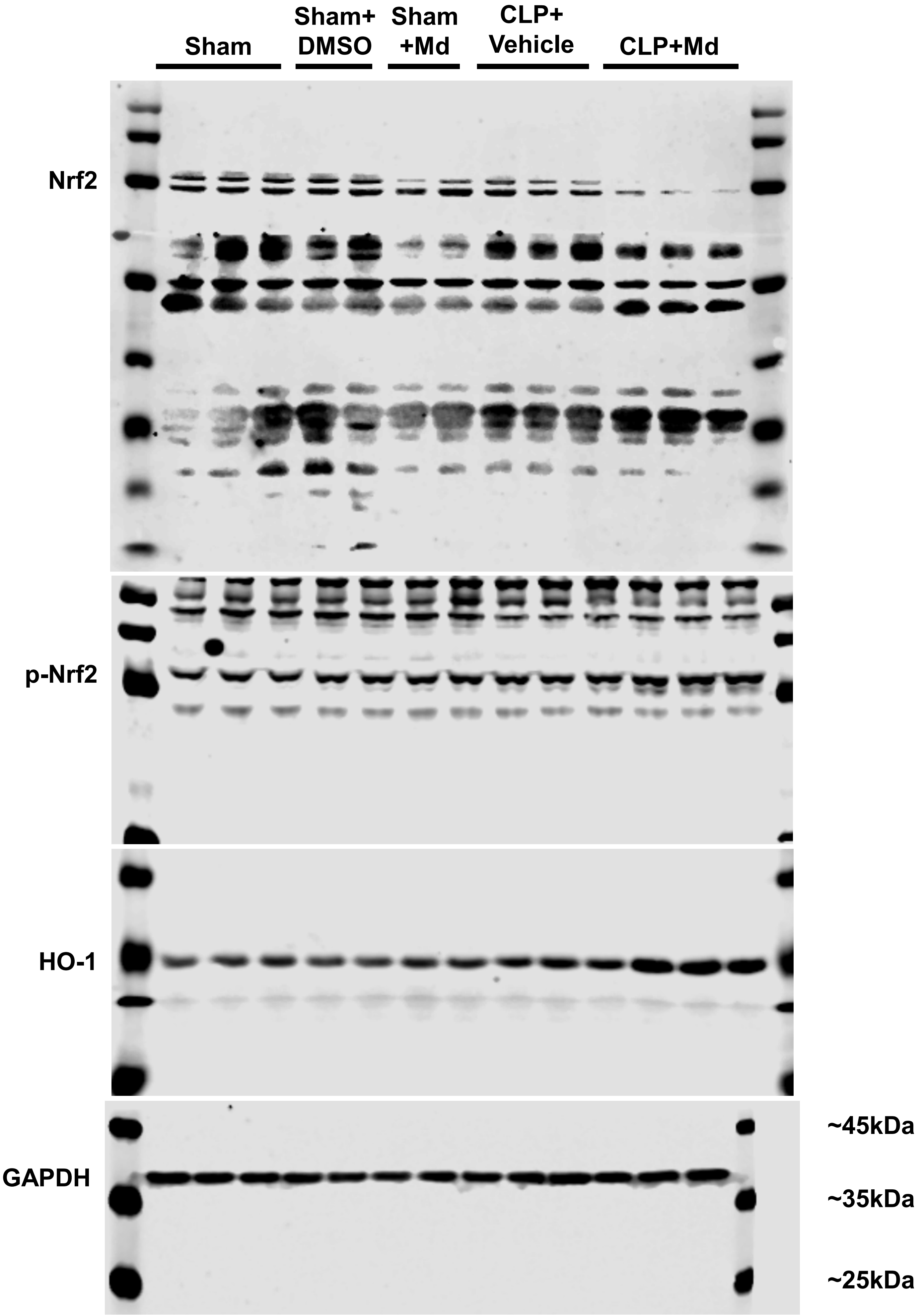

Full unedited gel/blot for Figure 4 J-3

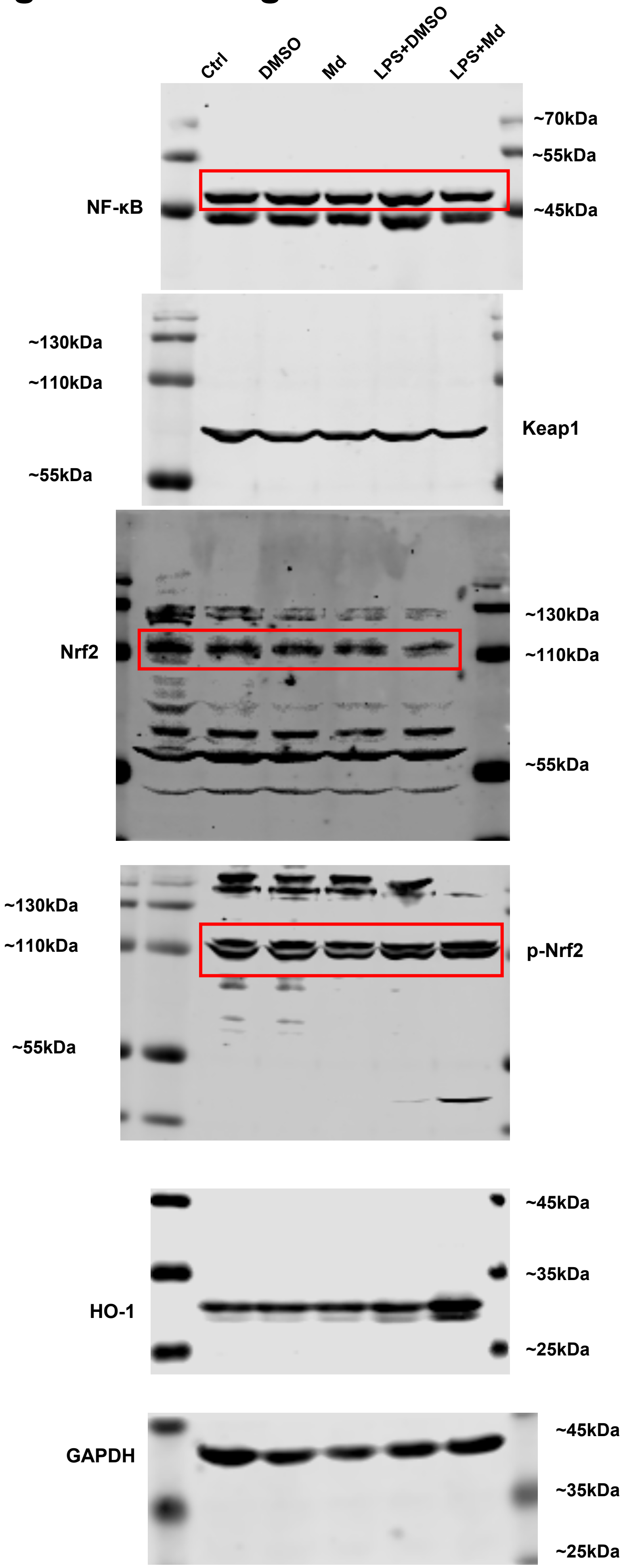

Full unedited gel/blot for Figure 5 C-1

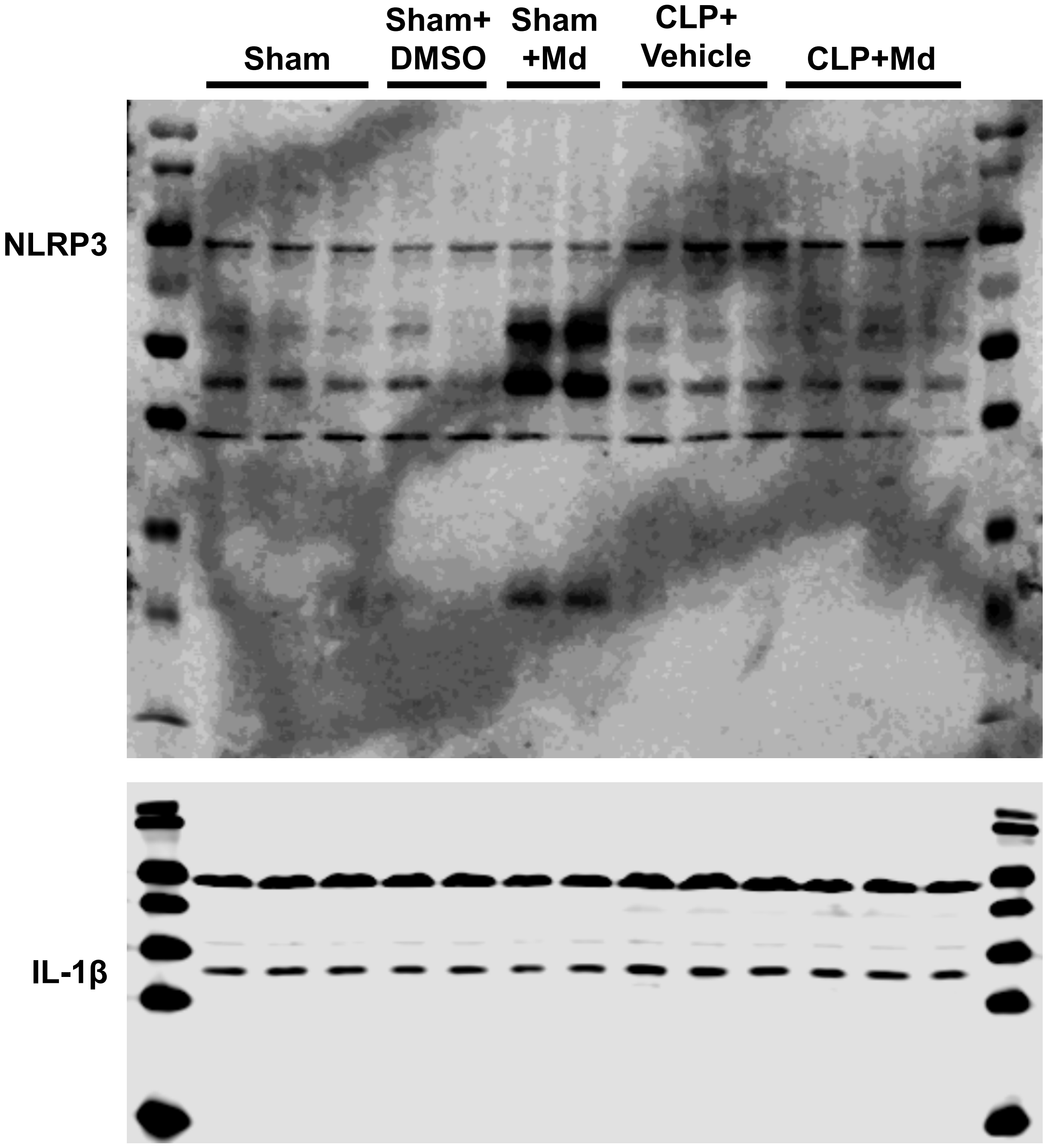

Full unedited gel/blot for Figure 5 C-2

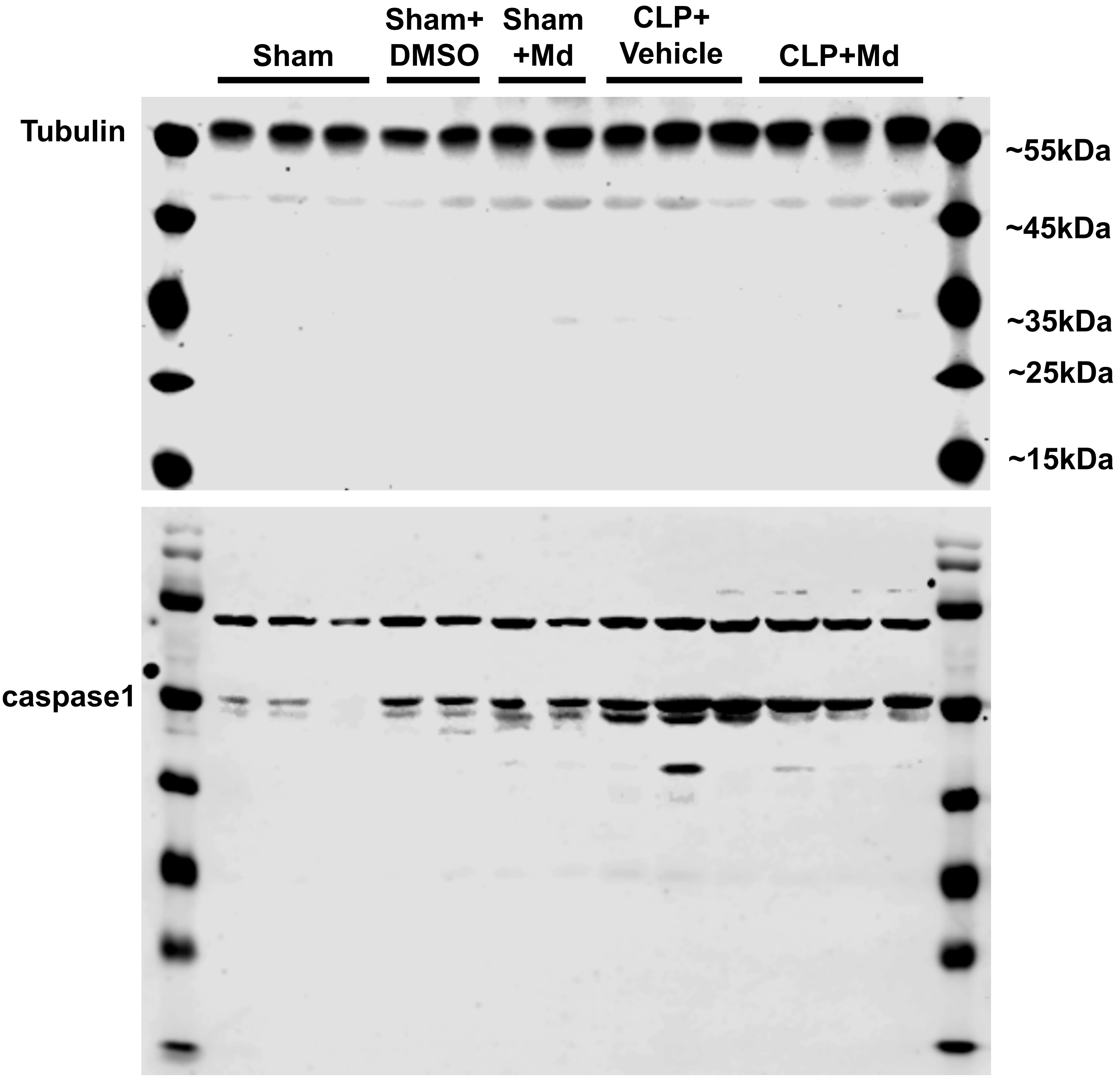

Full unedited gel/blot for Figure 5 C-3

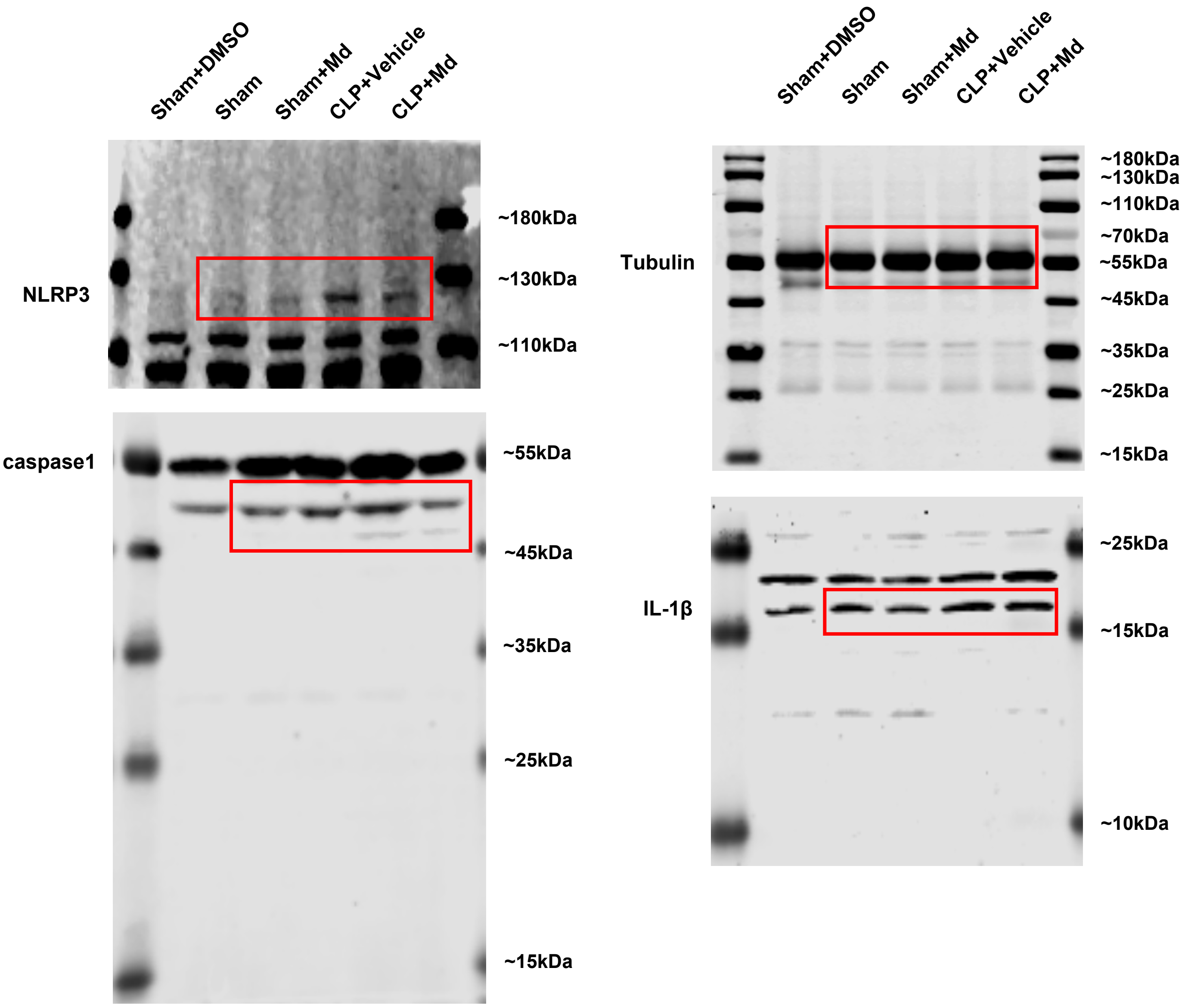

Full unedited gel/blot for Figure 5 E-1

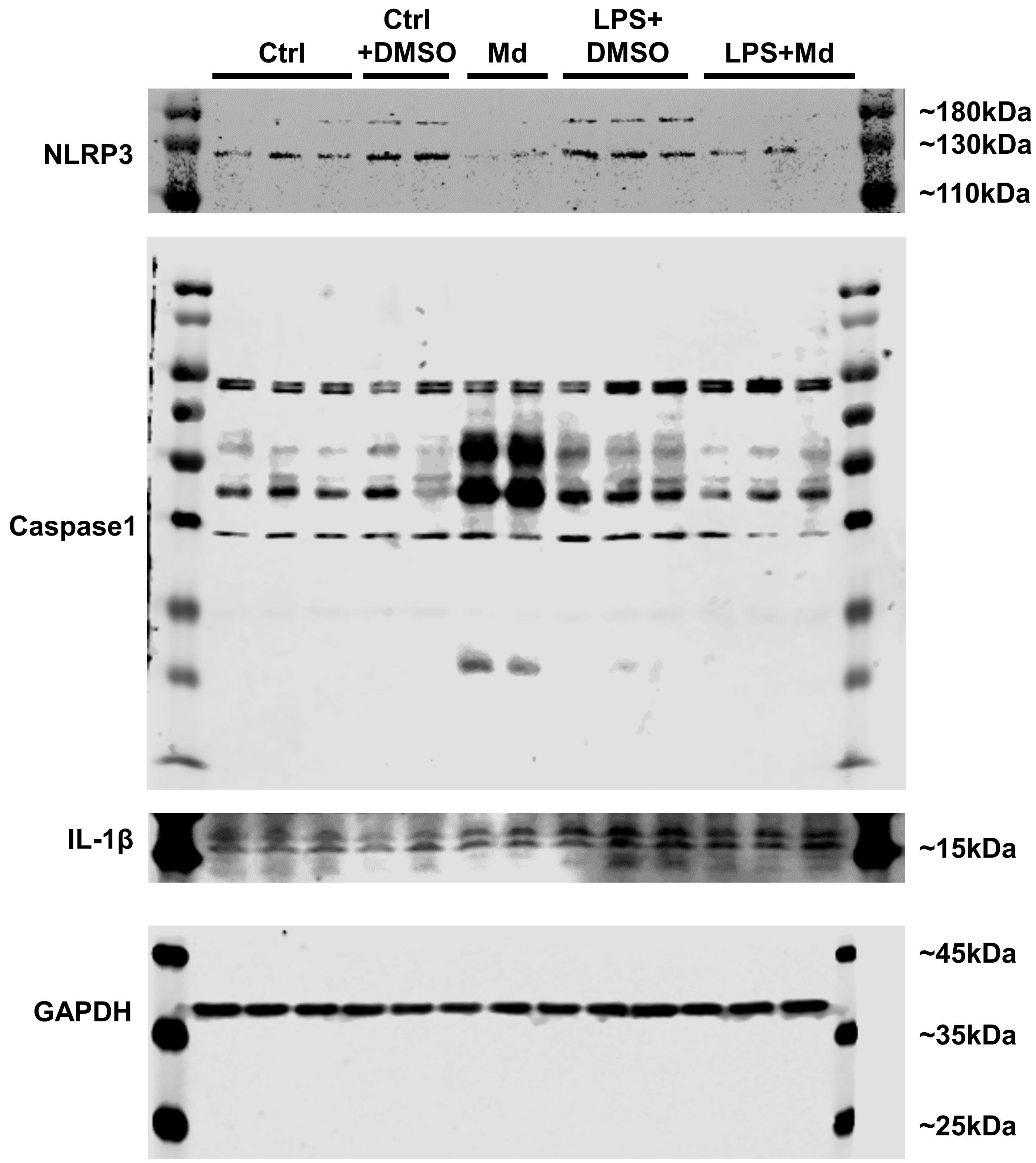

Full unedited gel/blot for Figure 5 E-2

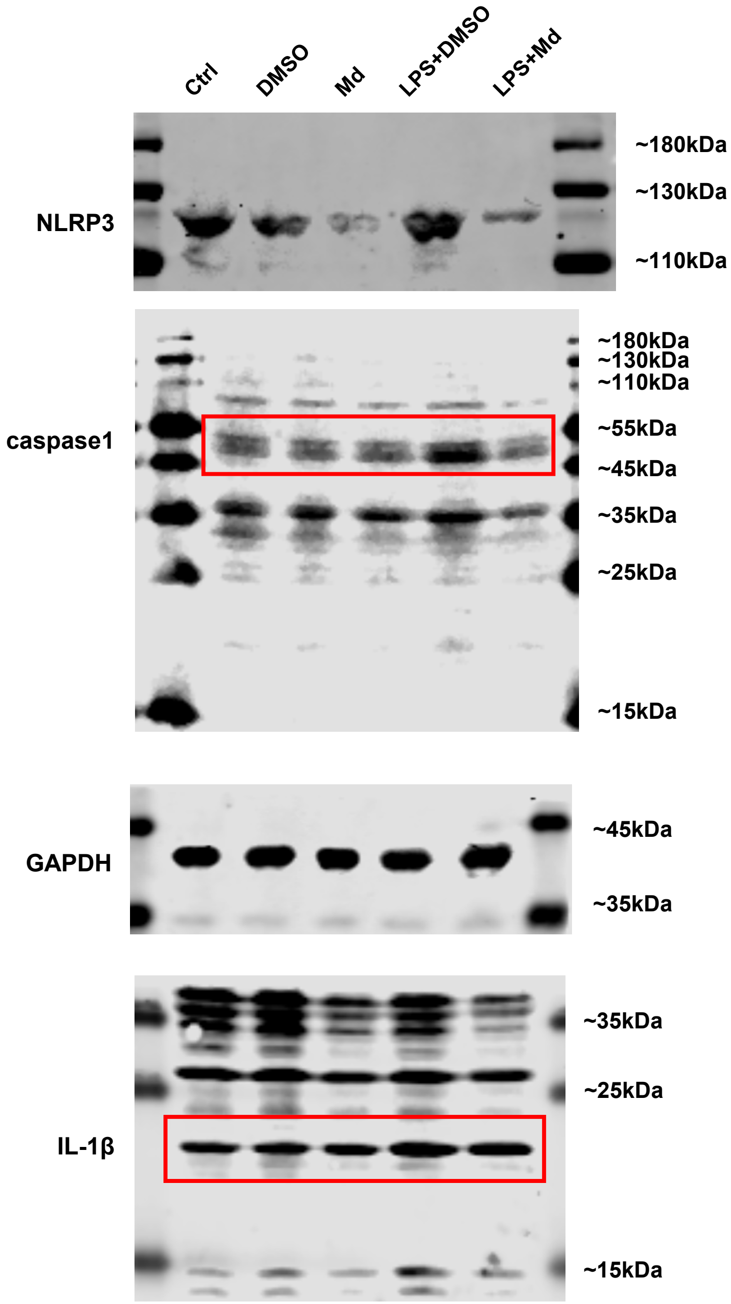

Full unedited gel/blot for Figure 6

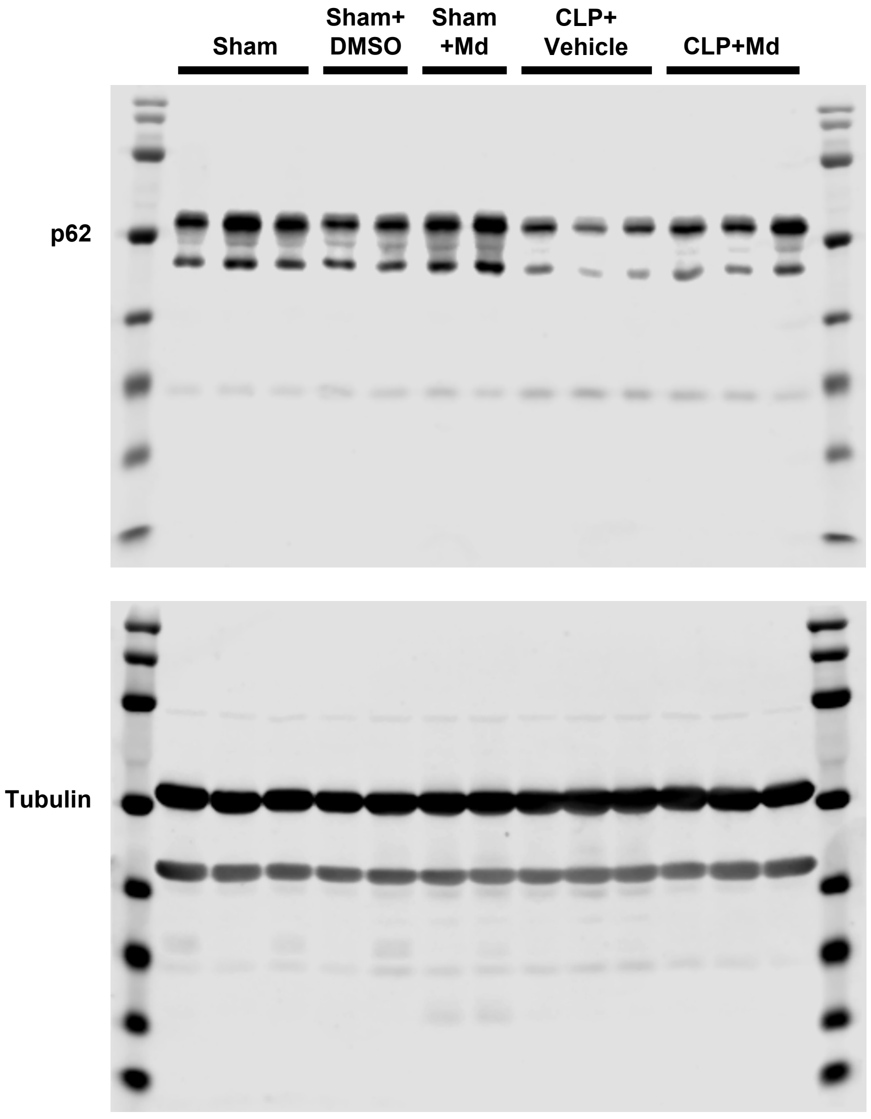

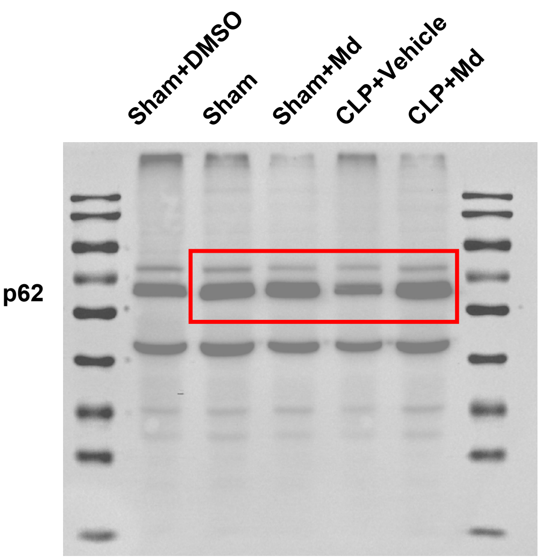

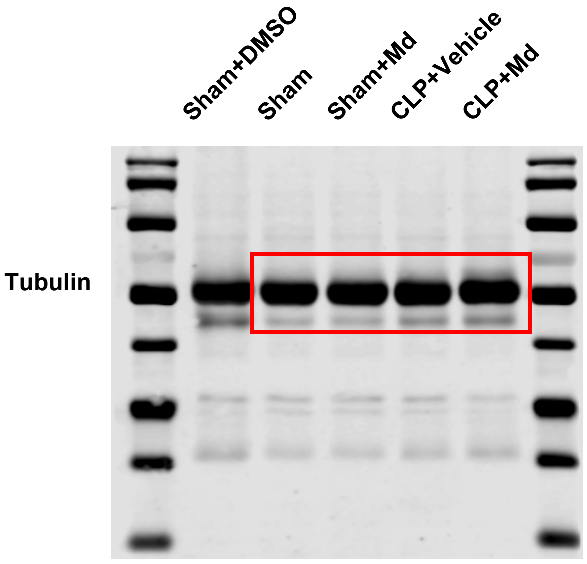

Full unedited gel/blot for Figure 6 E-1

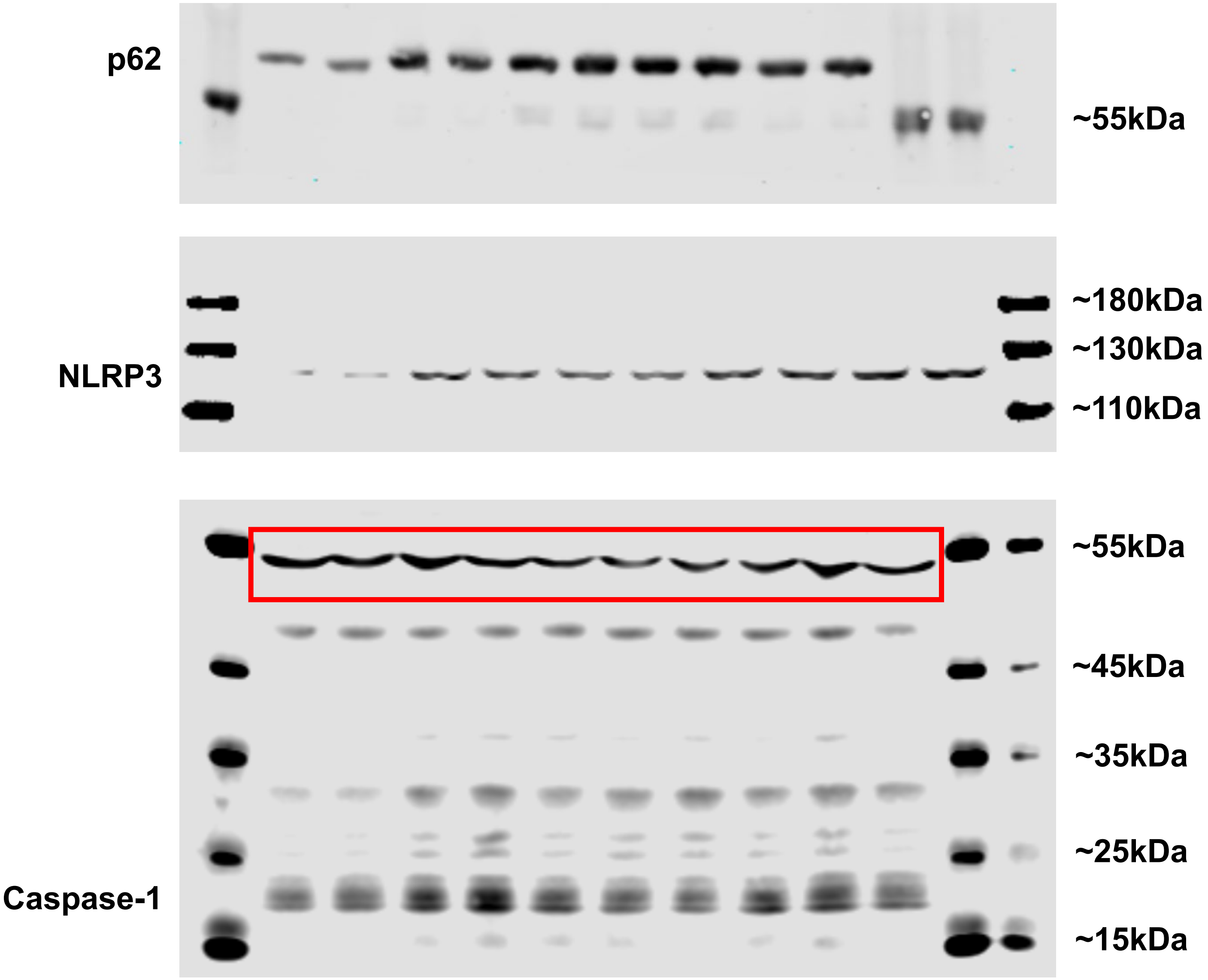

Full unedited gel/blot for Figure 6 E-2

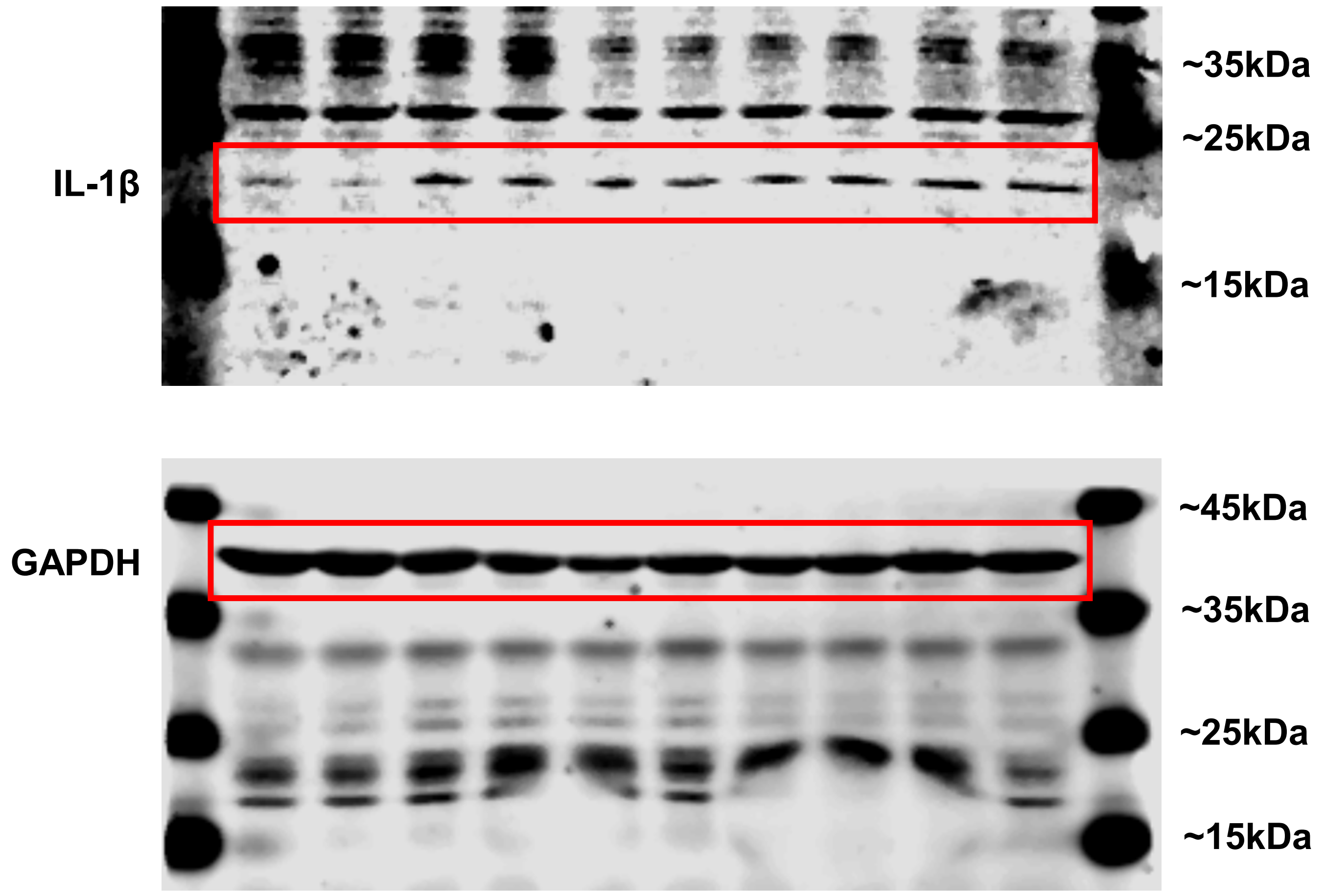

Full unedited gel/blot for Figure 6 H

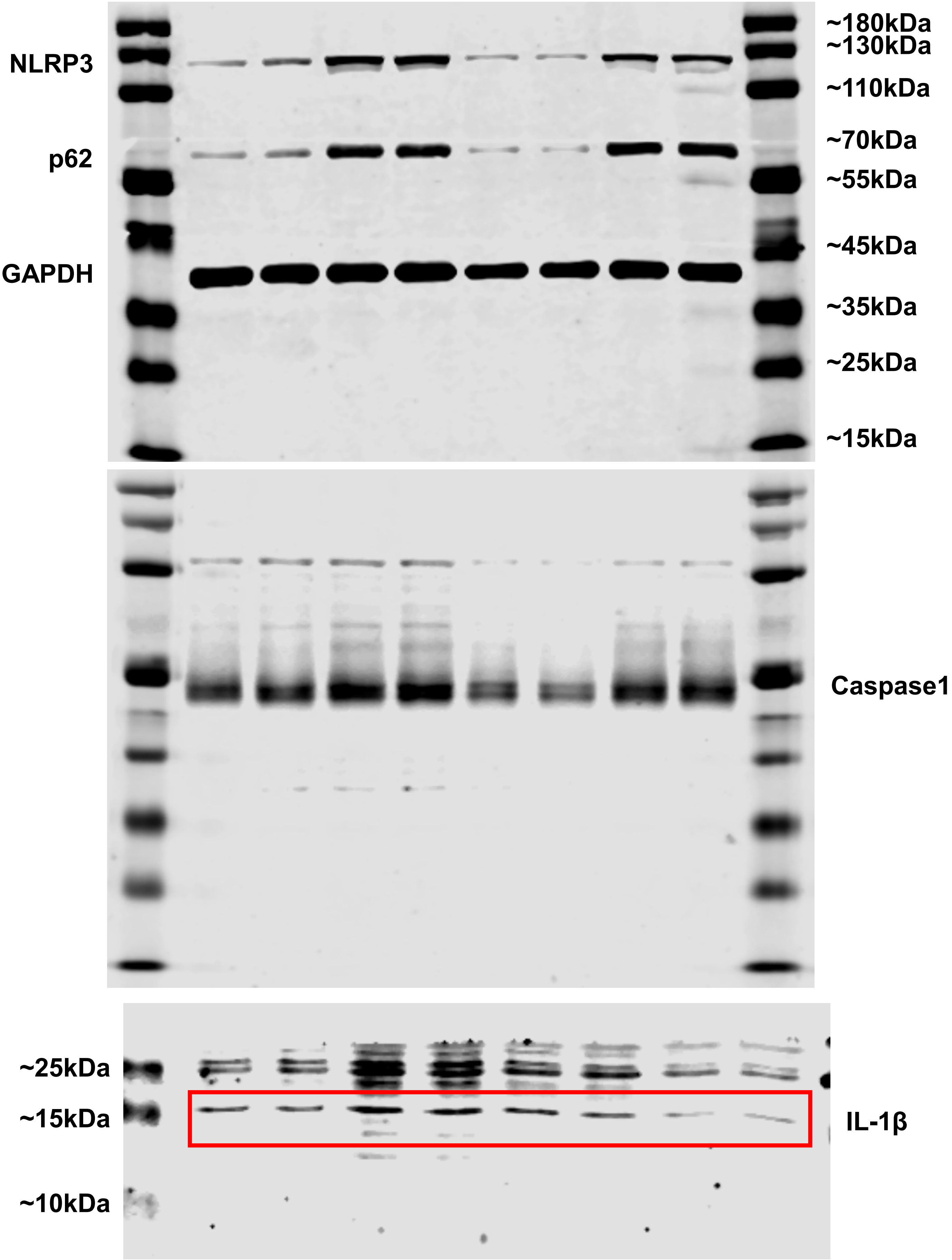

Full unedited gel/blot for Supplementary Figure 4 A

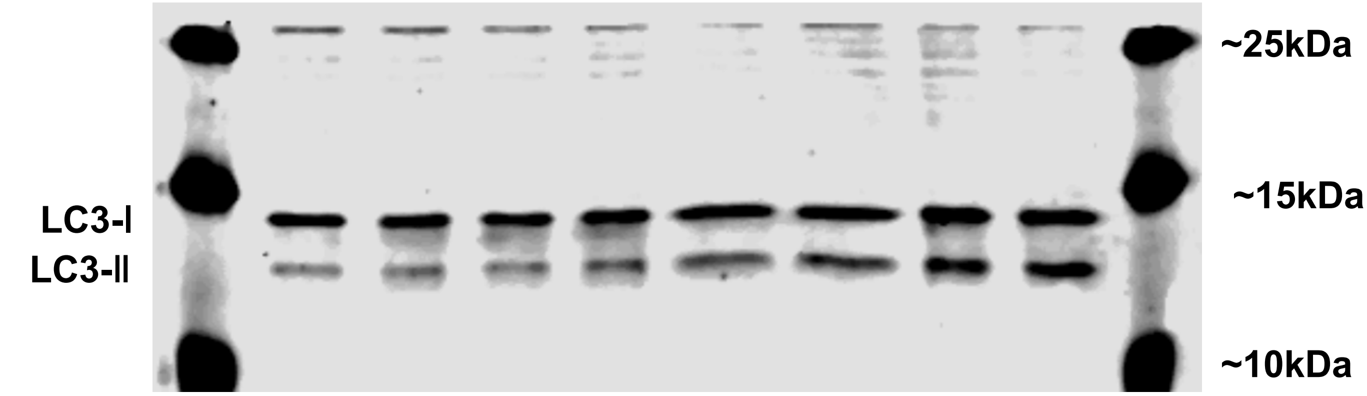

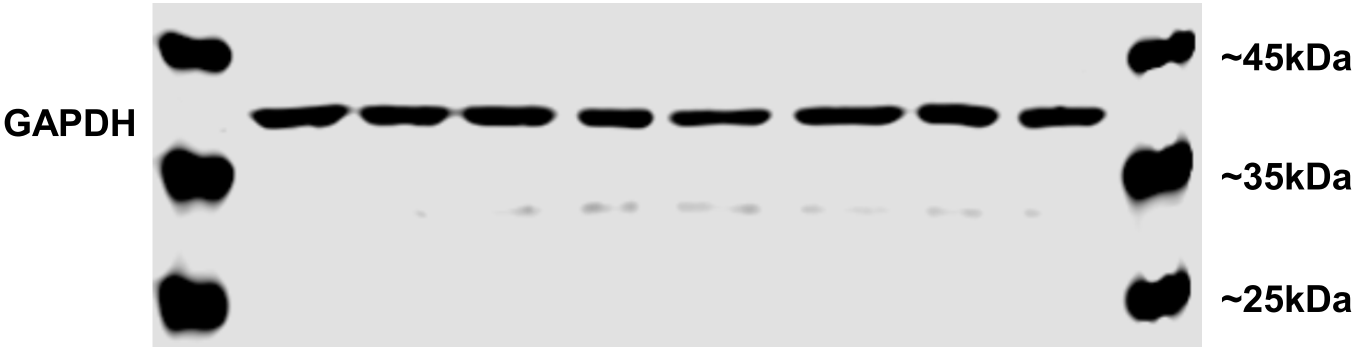

Full unedited gel/blot for Supplementary Figure 4 C-1

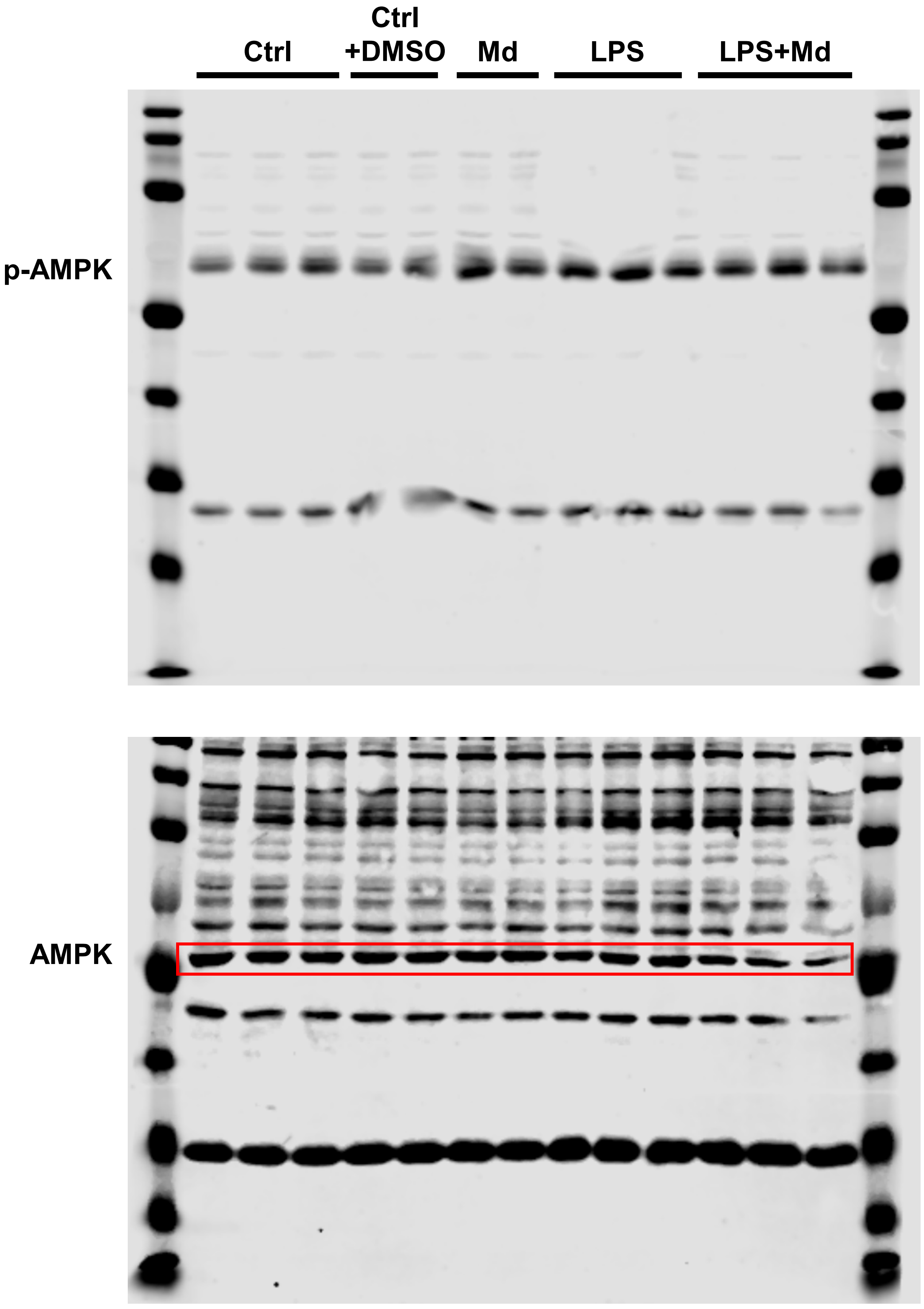

Full unedited gel/blot for Supplementary Figure 4 C-2

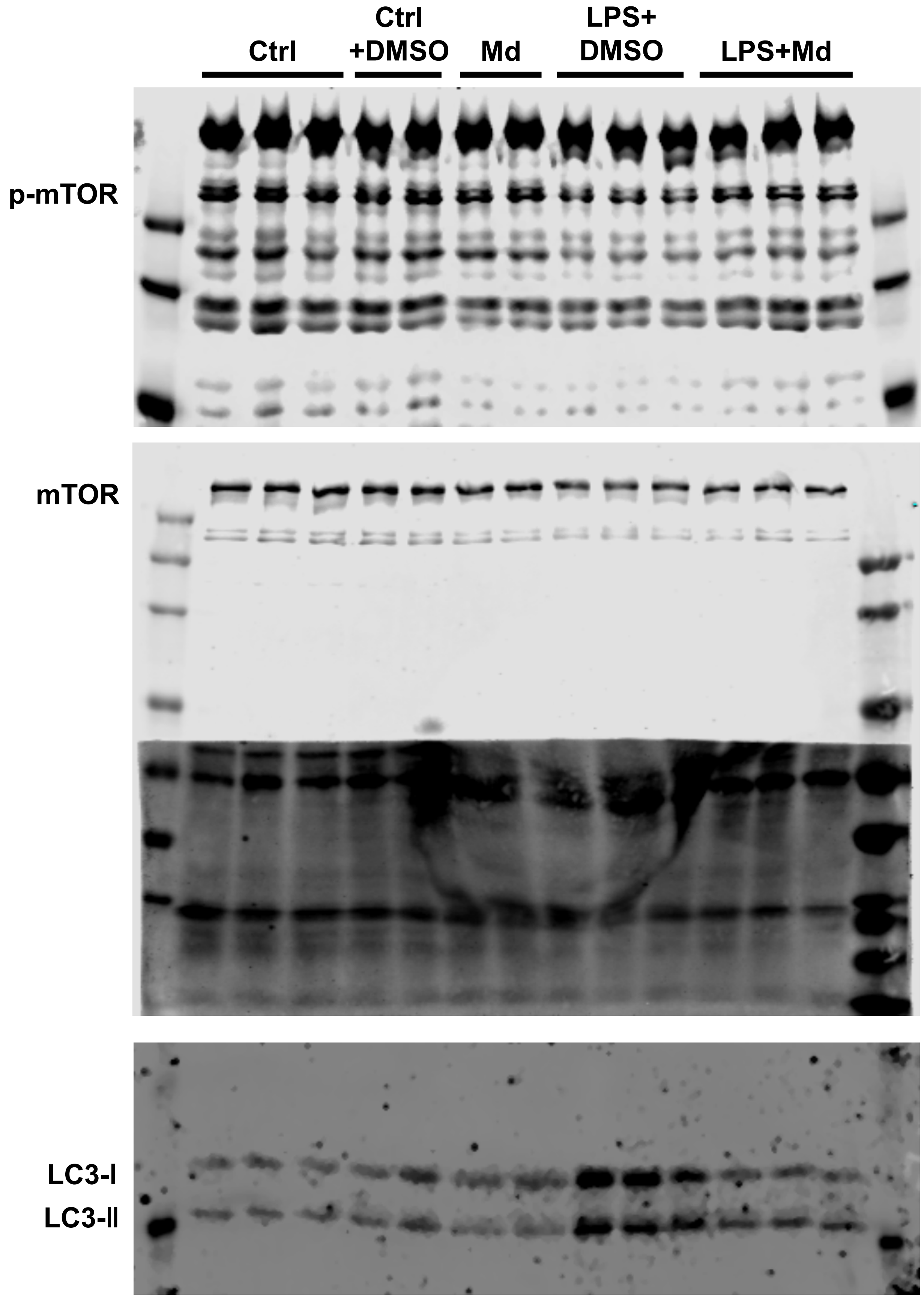

Full unedited gel/blot for Supplementary Figure 4 C-3

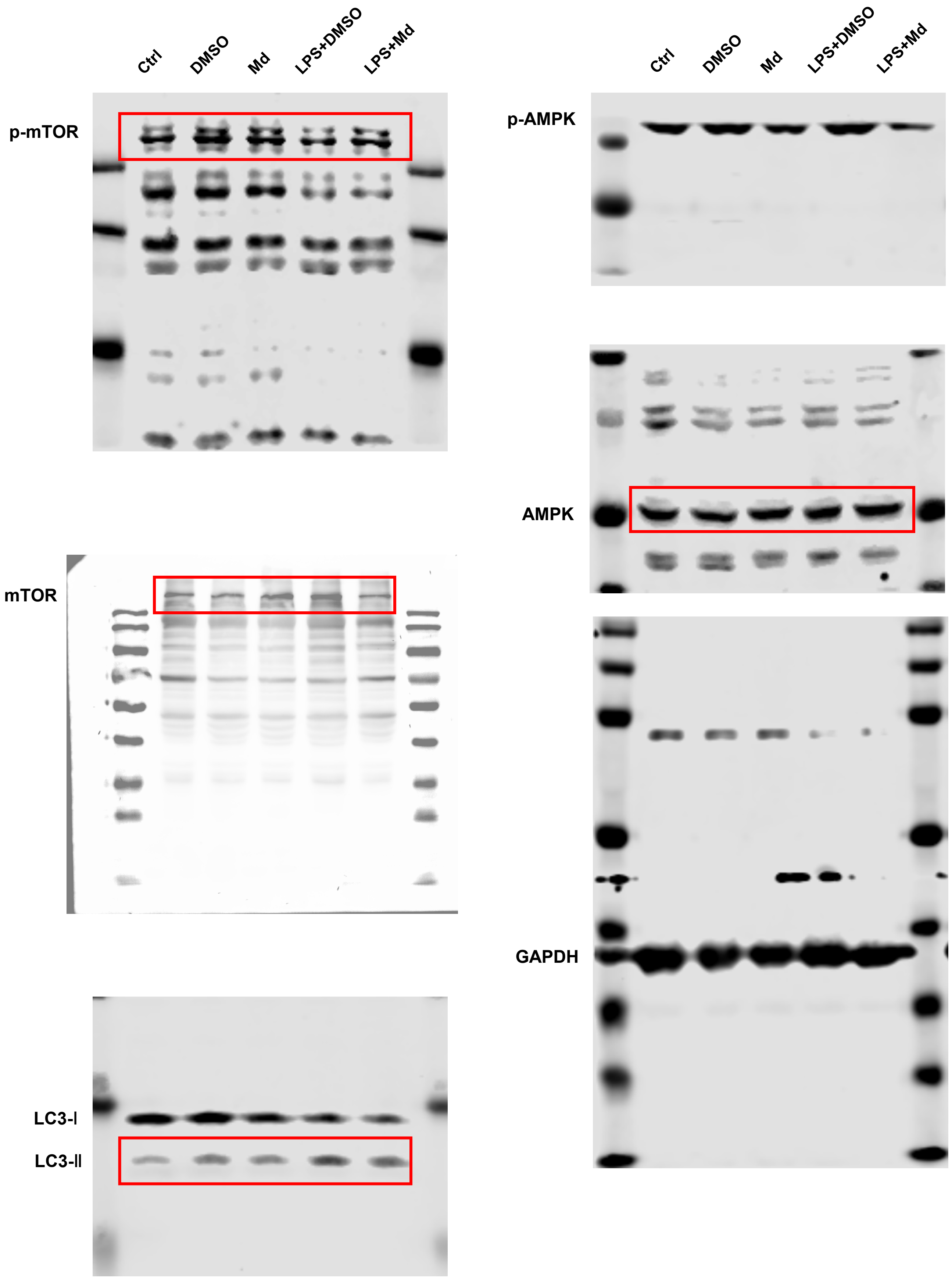

Full unedited gel/blot for Supplementary Figure 4 E-1

Ctrl | Ctrl +DMSO | Md | LPS+ DMSO | LPS+Md

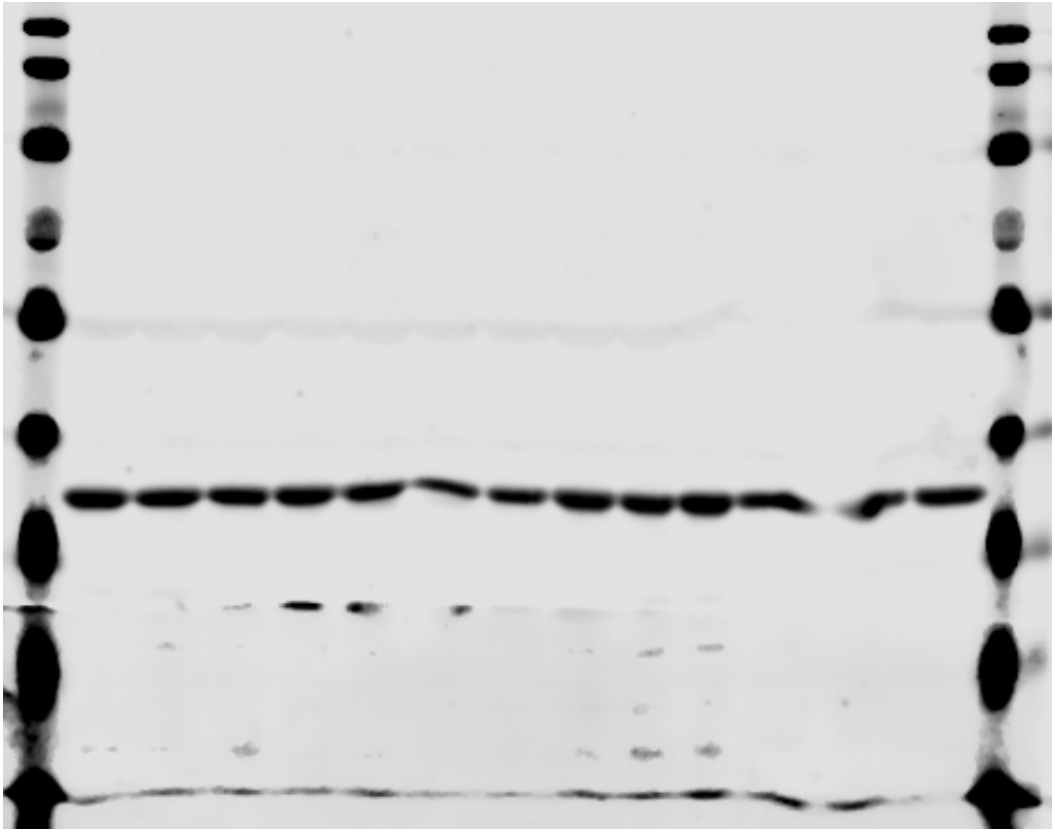

FUNDC1

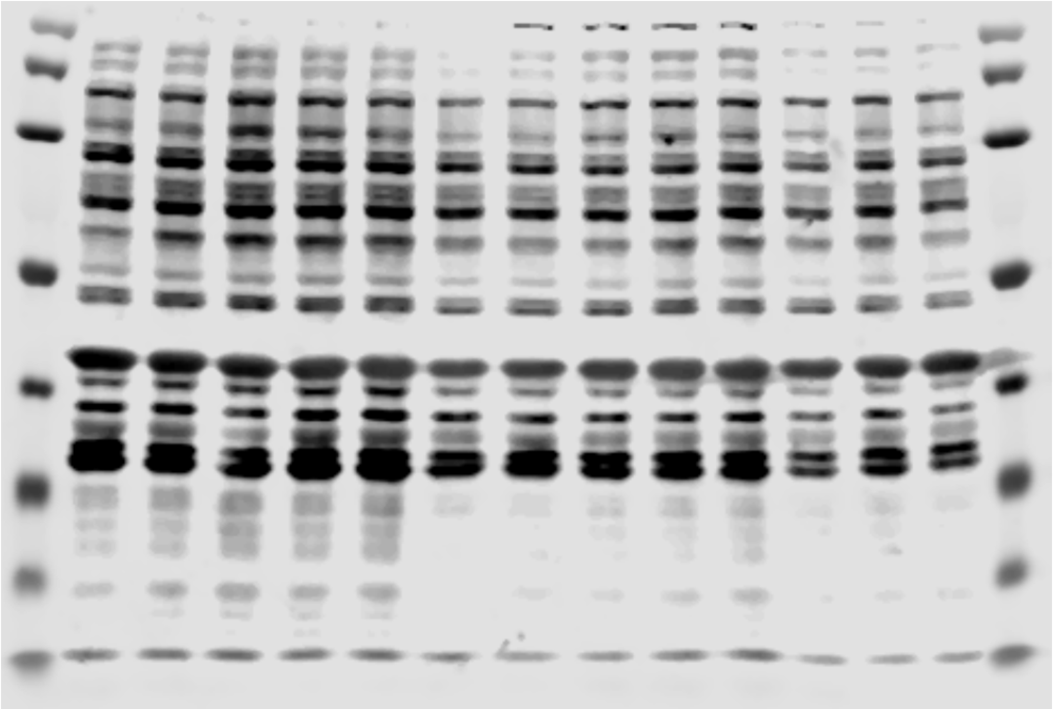

PINK1

Full unedited gel/blot for Supplementary Figure 4 E-2

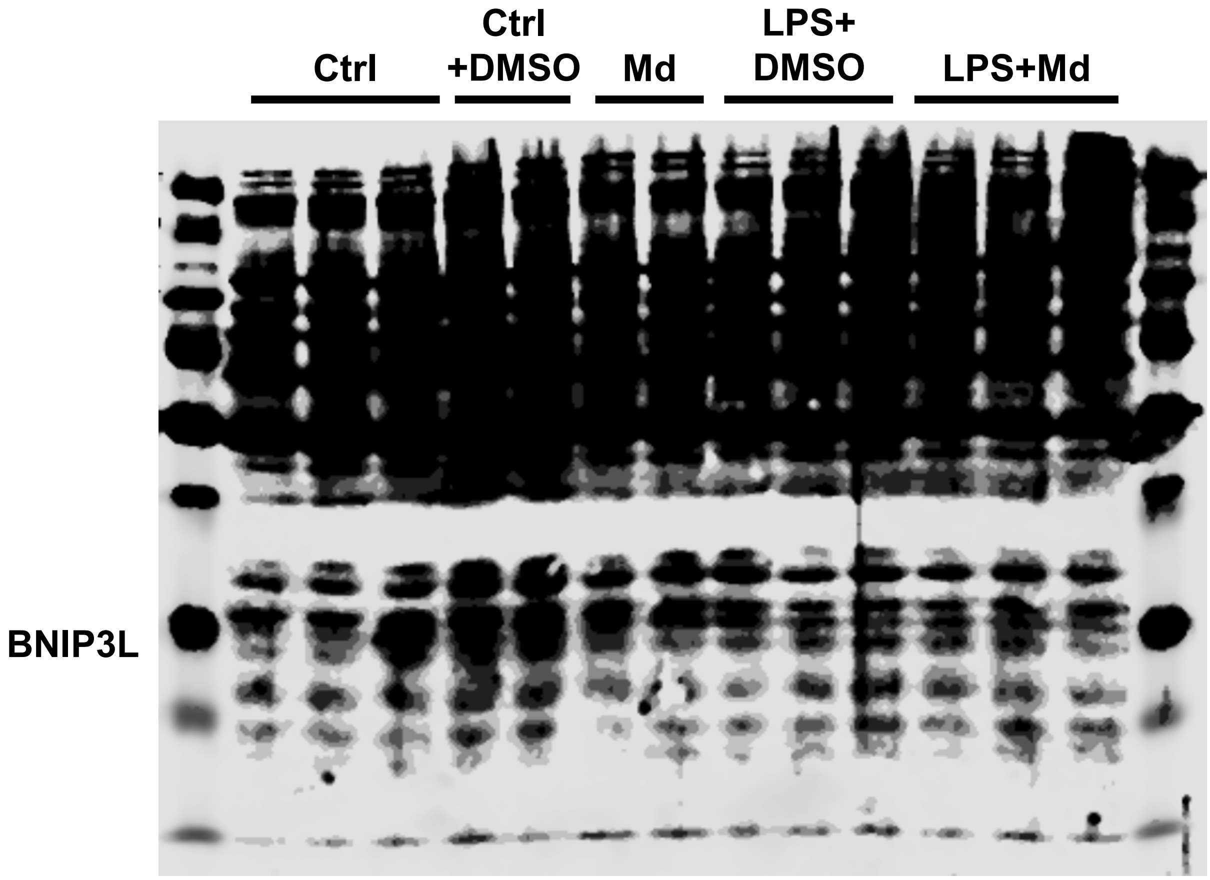

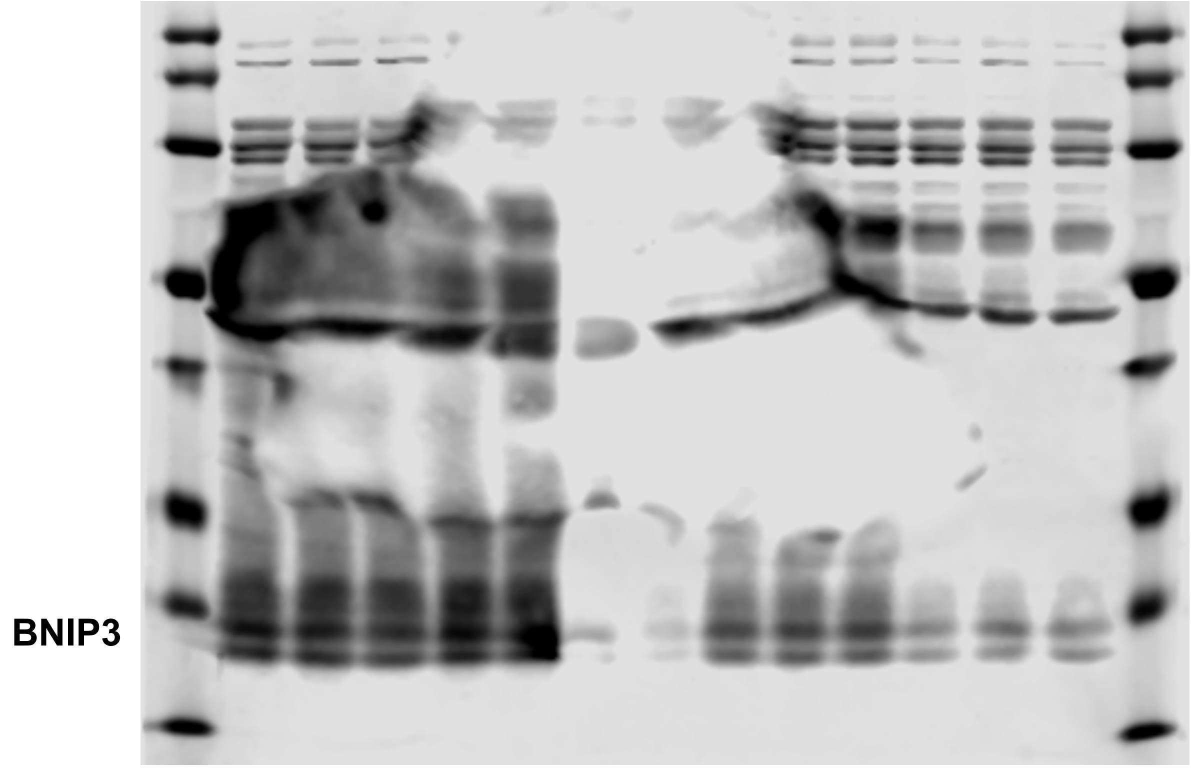

Full unedited gel/blot for Supplementary Figure 4 E-3

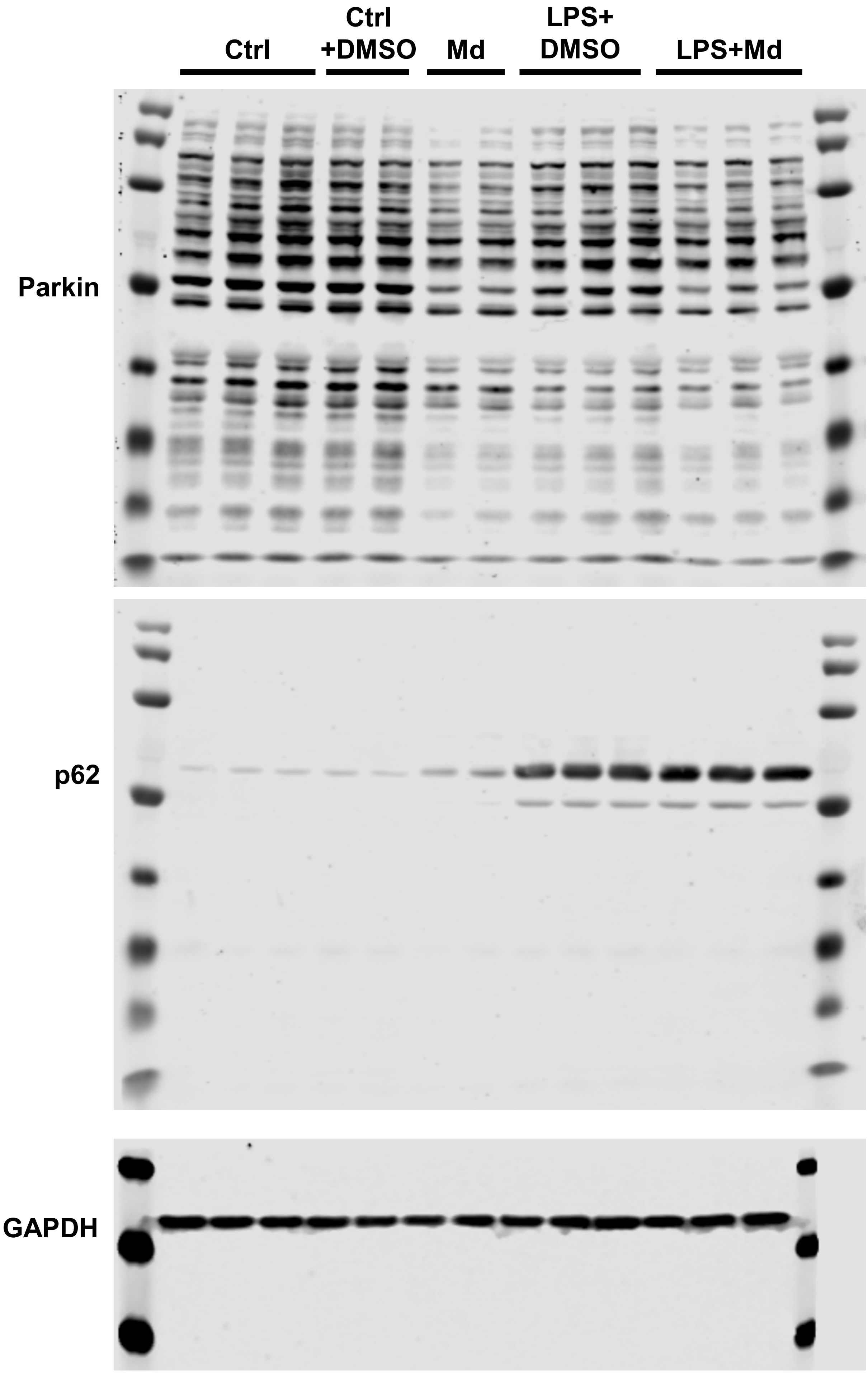

Full unedited gel/blot for Supplementary Figure 4 E-4

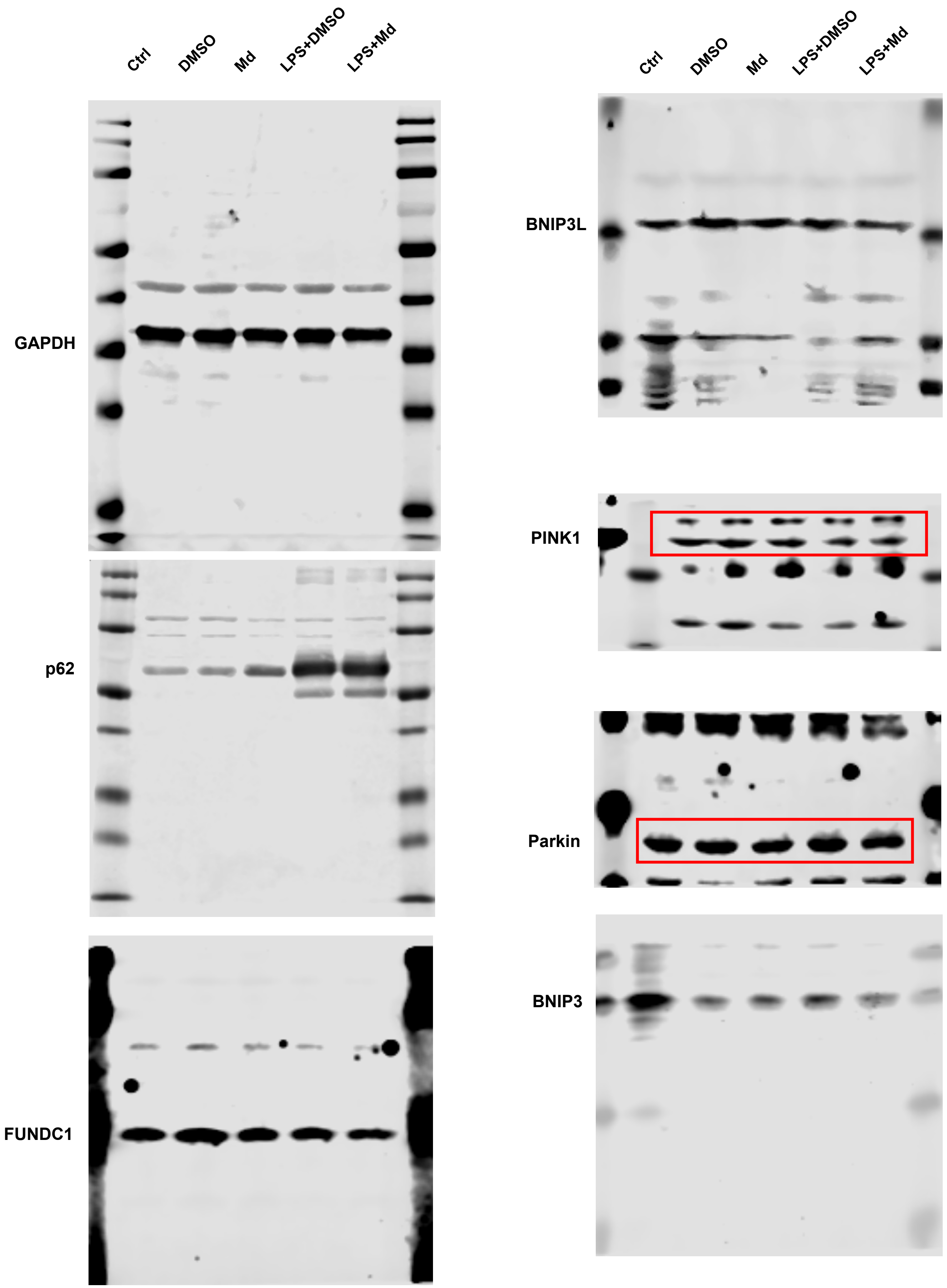

Supplement: Supplementary file 1 — Data S1. [file CNS-31-e70149-s001.pdf]
