# Supplementary material for: Protective Effects of Mdivi‐1 on Cognition Disturbance Following Sepsis in Mice via Alleviating Microglia Activation and Polarization
Source: CNS Neurosci Ther. 2025 Jan 10;31(1):e70149. doi: 10.1111/cns.70149 (PMC11719124; doi:10.1111/cns.70149)
Supplement: Supplementary file 2 — Appendix S1. [file CNS-31-e70149-s002.docx]

**Supplementary data**


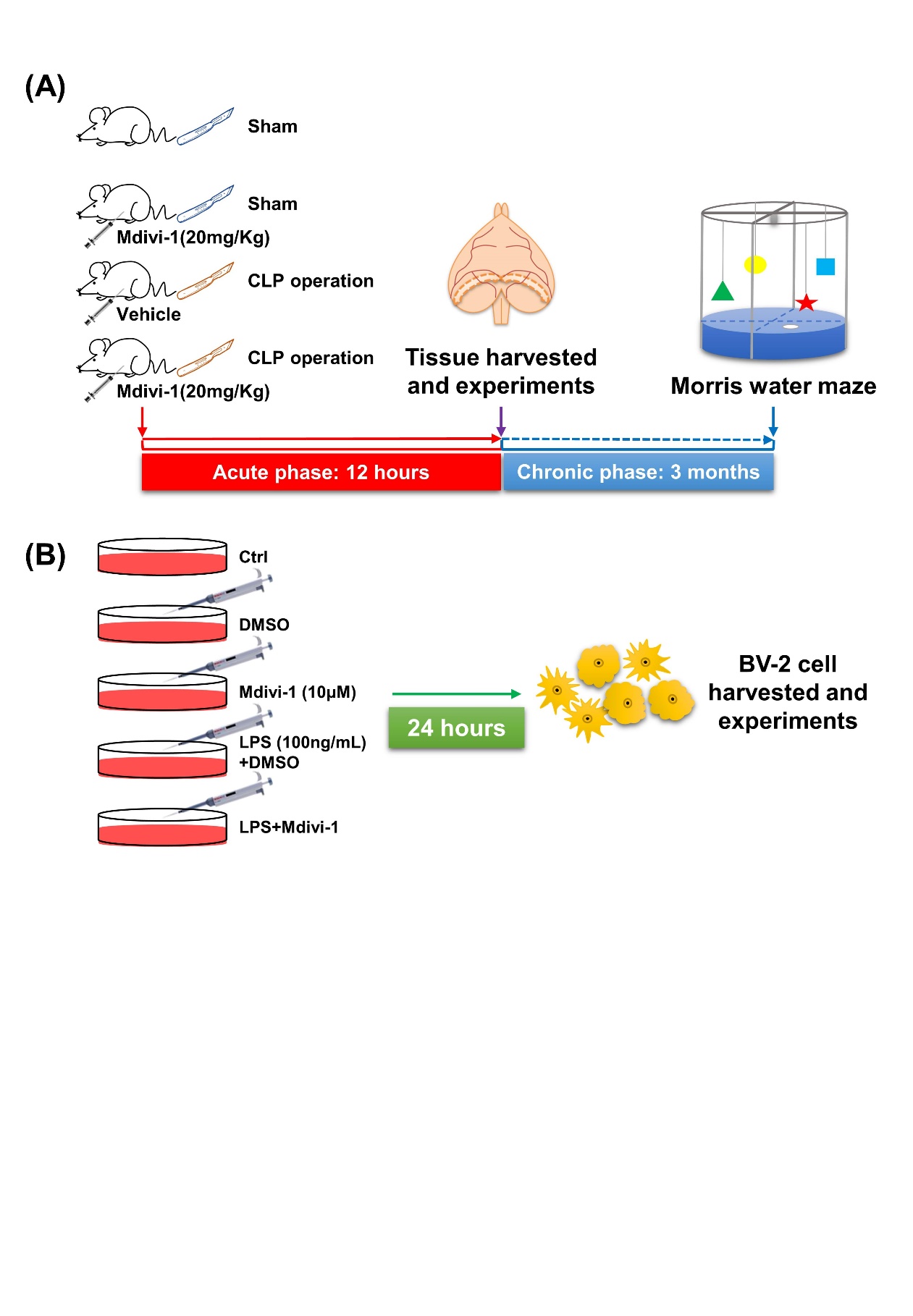


**Supplementary Figure 1 Strategies of experiments.**

A. 8-week-old female C57BL/6J mice were randomly divided into four groups: Sham group (Sham), Sham+Mdivi-1 group (Sham+Md), CLP surgery control group (CLP+Vehicle) and CLP surgery+Mdivi-1 group (CLP+Md). Mdivi-1 was dissolved in DMSO at a final concentration of 10mg/mL and intraperitoneal injected in the dose of 20mg/kg. The survivors were used to conduct Morris’s water maze three months later. The hippocampus was harvested 12 hours later or after the animal behavior test for the experimental use of acute and chronic phase of SAE, respectively. B. BV2 cell was randomly divided into five groups: Control group (Ctrl), DMSO group (DMSO), Mdivi-1 group (Md), LPS+DMSO group (LPS+DMSO) and LPS+Mdivi-1 group (LPS+Md). LPS and Midivi-1 were given in the dose of 100ng/mL and 10μM, respectively. Cells were harvested 24 hours later for experimental use.


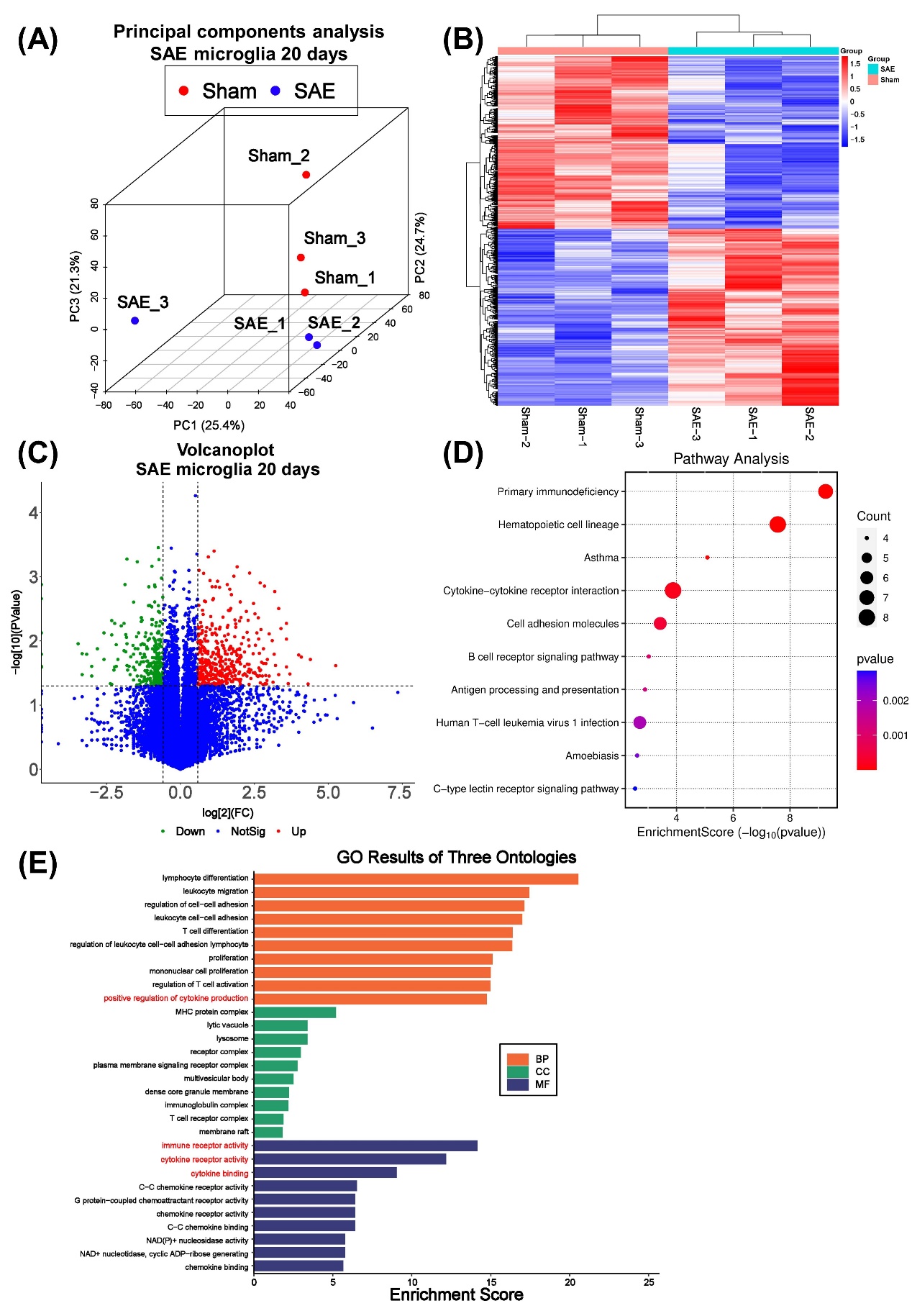


**Supplementary Figure 2 Microglia remain activated after 20 days following induction of polymicrobial sepsis *in vivo*.**

A. Principal component analysis of gene expression in microglia after 20 days of sepsis (n=3). B. The differentially expressed genes were displayed in a heat map of microglia after 20 days of sepsis. C. The volcanic map with gene tagging in microglia after 20 days of sepsis. D. The Pathway enrichment analysis of the microglia after 20 days of sepsis. E. The Gene Ontology analysis of biological process of the microglia after 20 days of sepsis.


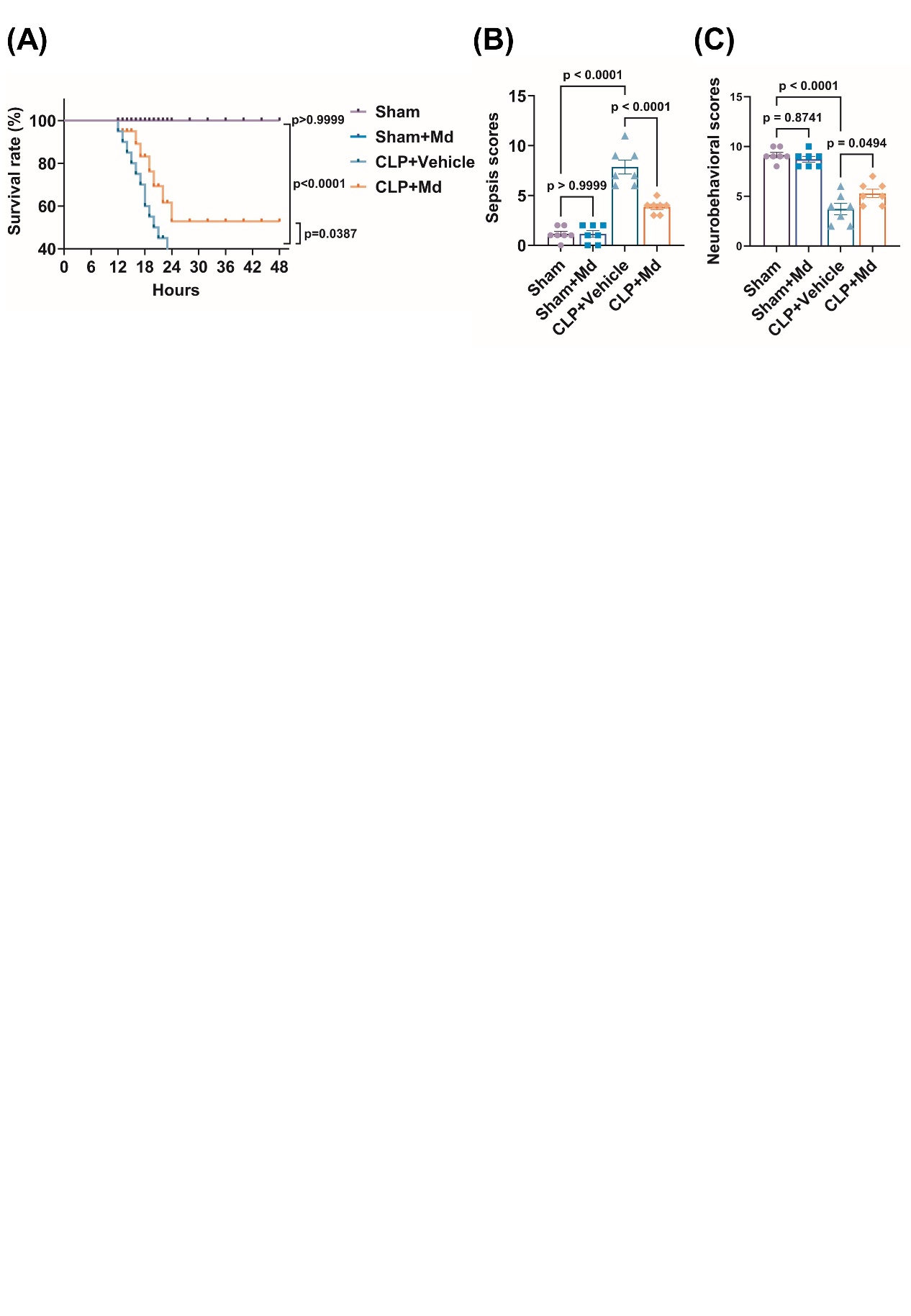


**Supplementary Figure 3 Survival rate, murine sepsis score (MSS), and** **neurobehavioral scores after** **CLP induction.**

A. Survival rate in 48 hours after CLP induction (F=31.93; p <0.0001). B. Murine sepsis score after 24 hours of CLP induction (F=53.85; p <0.0001). C. Neurobehavioral scores after 24 hours of CLP induction (F=43.20; p <0.0001).


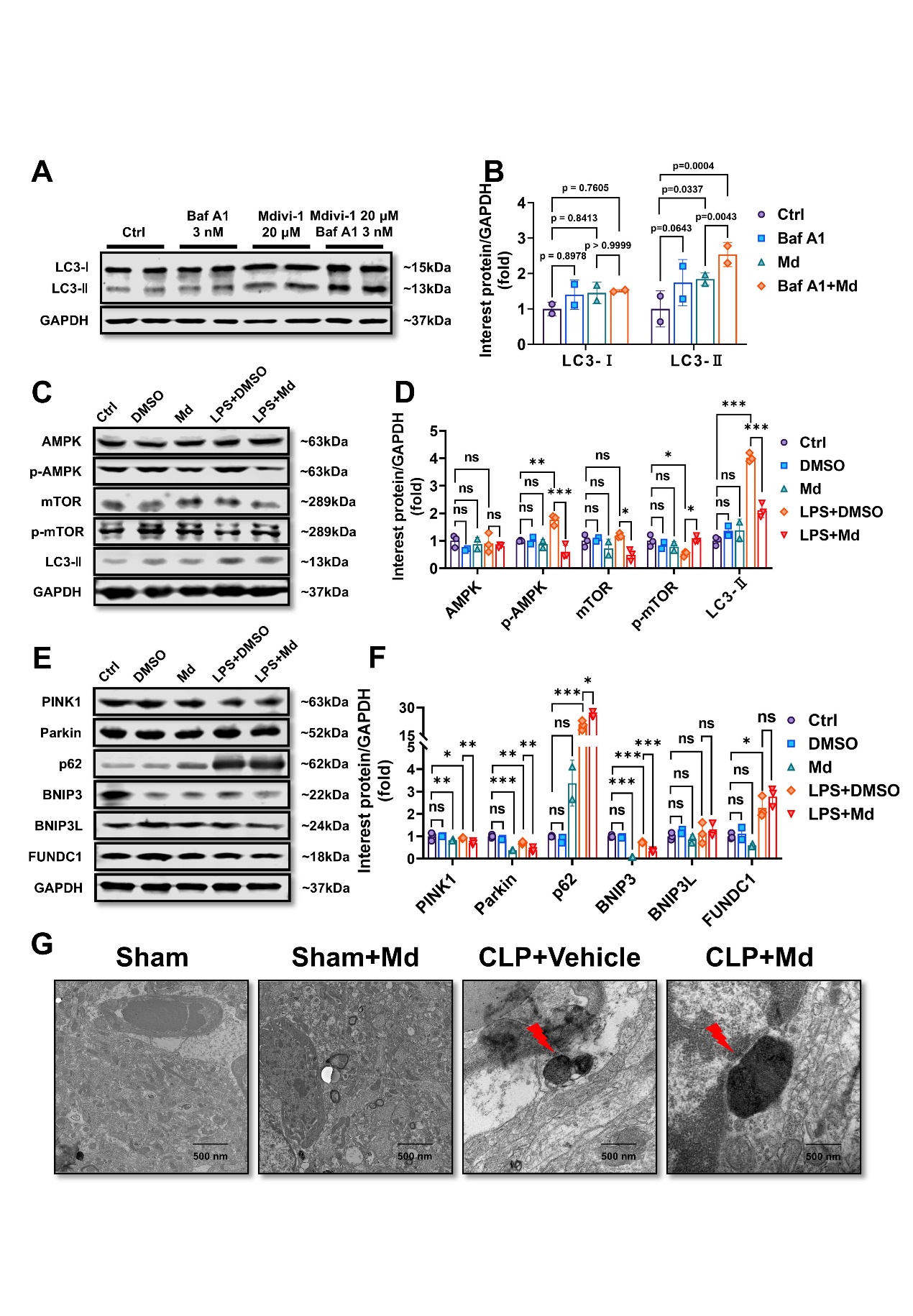


**Supplementary Figure 4 The effects of Mdivi-1 on autophagy and mitophagy in LPS-induced BV2 cell model.**

A-B. Representative Western blots (A) and quantitative analysis (B) of LC3-Ⅰ and LC3-Ⅱ of BV2 cell in autophagy flow detection test (F=48.9; p<0.0001). C-D. Representative Western blots (C) and quantitative analysis (D) of AMPK, p-AMPK, mTOR, p-mTOR and LC3-Ⅱ levels in LPS-induced BV2 cell model (F=1.419; p<0.0001). E-F Representative Western blots (E) and quantitative analysis (F) of PINK1, Parkin, p62, BNIP3, BNIP3L and FUNDC1 levels in LPS-induced BV2 cell model (F=175.9; p<0.0001). G. Representative images scanning by transmission electron microscope of hippocampal autophagosome formation in SAE mice after 12 hours (Scale bar=500nm). A-B. n=3 per group. C-F. Ctrl group (n=3 per group), DMSO group (n=2 per group), Md group (n=2 per group), LPS+DMSO group (n=3 per group), LPS+Md group (n=3 per group). G. n=2 per group. All data were shown as mean ± SD.


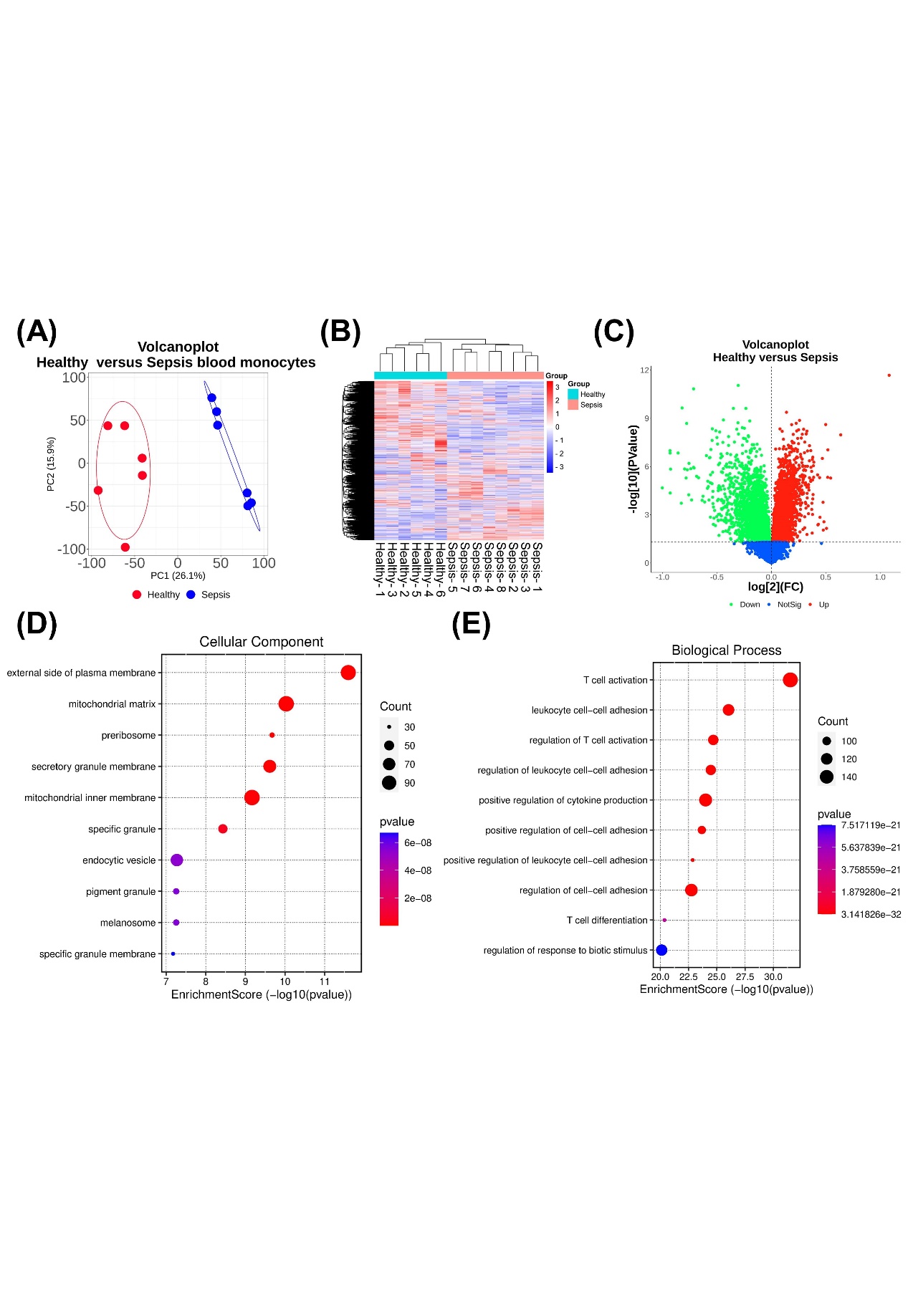


**Supplementary Figure 5 Peripheral blood mononuclear-macrophages exhibit excessive mitochondrial division and inflammation in sepsis patients.**

A. Principal component analysis of gene expression in the peripheral blood monocytes in healthy and sepsis patients (n=6). B. The differentially expressed genes are displayed in a heat map of the peripheral blood monocytes in healthy and sepsis patients. C. The volcanic map with gene tagging in the peripheral blood monocytes in healthy and sepsis patients. D. The cluster analysis results of cellar component from the peripheral blood mononuclear-macrophages in healthy and sepsis patients. E. The cluster analysis results of biological process from the peripheral blood mononuclear-macrophages in healthy and sepsis patients.


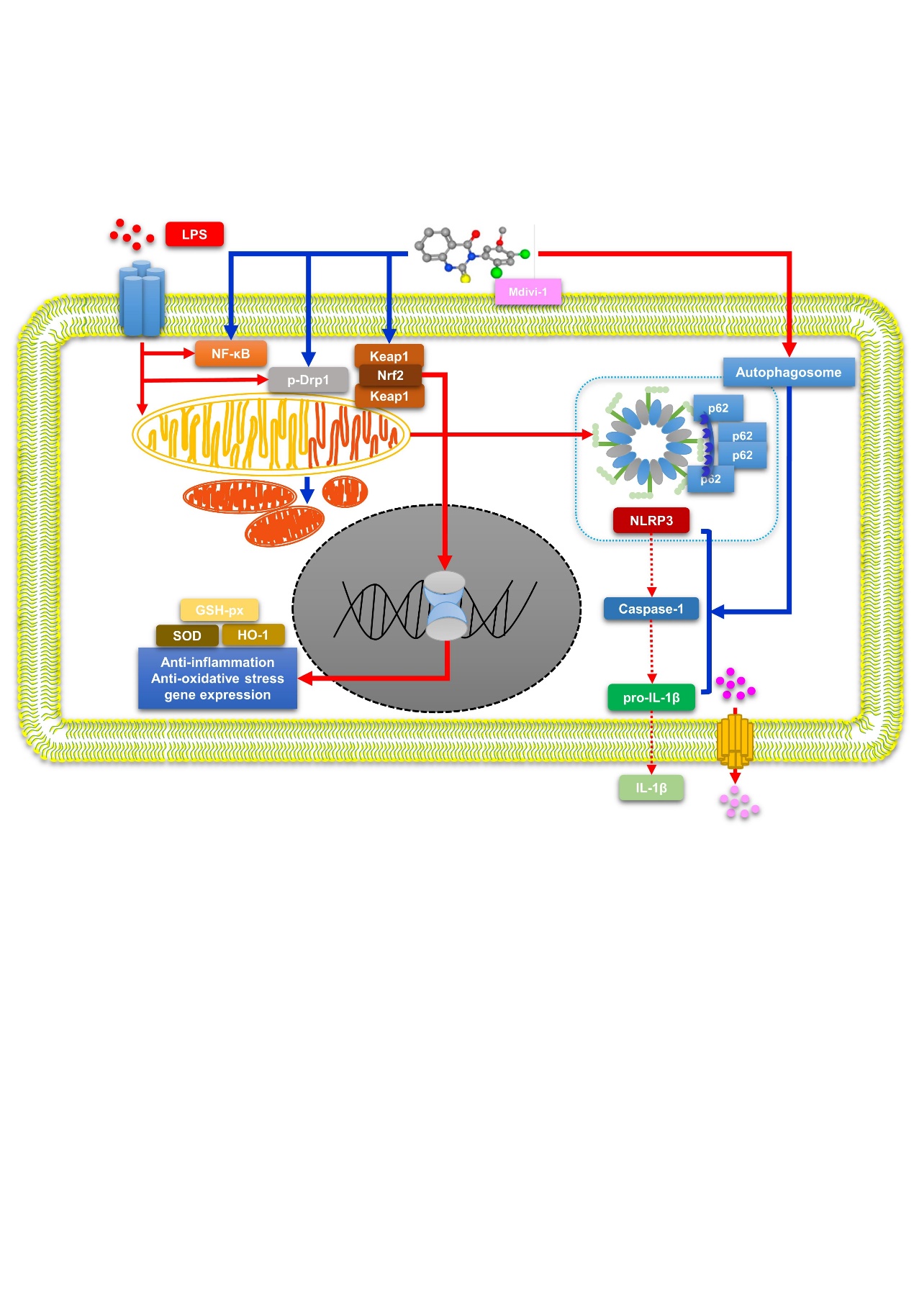


**Supplementary Figure 6 Schematic representation of the Mdivi-1 on microglia in SAE.**

| **Supplementary Table 1 Primer sets used in the study** | | |
| --- | --- | --- |
| Gene | Forward primer (5'-3') | Reversed primer (5'-3') |
| GAPDH | GGGTCCCAGCTTAGGTTCAT | CCCAATACGGCCAAATCCGT |
| IL-1β | TCCAGGATGAGGACATGAGCAC | GAACGTCACACACCAGCAGGTTA |
| IL-6 | CCACTTCACAAGTCGGAGGCTTA | GCAAGTGCATCATCGTTGTTCATAC |
| IL-10 | CTTACTGACTGGCATGAGGATCA | GCAGCTCTAGGAGCATGTGG |
| TNF-α | AAGCCTGTAGCCCACGTCGTA | GGCACCACTAGTTGGTTGTCTTTG |
| TGF-β1 | ACTGGAGTTGTACGGCAGTG | GGGGCTGATCCCGTTGATT |
| p62 | ACTGCTCAGGAGGAGACGAT | CCGGGGATCAGCCTCTGTAG |

| **Supplementary Table 2 Antibodies used in the Western blots of this study** | | | | | |  |
| --- | --- | --- | --- | --- | --- | --- |
| **Abcam** | | Parkin | ab77924 | p-mTOR | 5536T |  |
| Drp1 | ab184247 | FUNDC1 | ab224722 | GAPDH | 5174S |  |
| Mff | ab241597 | caspase-1 | ab138483 | **GeneTex** | |  |
| Fis1 | ab71498 | **Cell Signaling Technology** | | Nrf2 | GTX103322 |  |
| OMA1 | ab154949 | p-Drp1 | 4494S | p-Nrf2 | GTX03209 |  |
| BDNF | ab108319 | PSD95 | 3450T | **Bioss** | |  |
| Iba-1 | ab283319 | Synaptophysin | 36406T | IL-1β | bs-0812R |  |
| NF-κB | ab32360 | GFAP | 3670T | **Biorbyt** | |  |
| Keap1 | ab139729 | Arg1 | 93668T | BNIP3 | orb4585 |  |
| iNOS | ab49999 | NLRP3 | 15101S | BNIP3L | orb101642 |  |
| mTOR | ab32028 | AMPK | 5831T | **Abclonal** | |  |
| p62 | ab109012 | p-AMPK | 2535S | MAP2 | A3278 |  |
| PINK1 | ab23707 | LC3AB | 12741S | HO-1 | A19062 |  |
| \| **Supplementary Table 3 Antibodies used in the Flow cytometry of this study** \| \| \| \| --- \| --- \| --- \| \| **BioLegend** \| \| \| PE/Cyanine7 anti-mouse CD11b \| 101215 \| \| Brilliant Violet 421^TM^ anti-mouse CD206 (MMR) \| 141717 \| \| FITC anti-mouse CD45 \| 103107 \| \| APC anti-mouse CD68 \| 137007 \| \| PE anti-mouse CD86 \| 105007 \| | | | | | | |
